# Accessing S_n≥2_ States of a TADF Emitter by Femtosecond NIR Spectroscopy

**DOI:** 10.1002/chem.70875

**Published:** 2026-03-23

**Authors:** Wiebke Haselbach, Jasmin Matthes, Andreas Prüfer, Simon L. Zimmermann, Monika Flörke, Thomas J. J. Müller, Peter Gilch, Barbara E. Nogueira de Faria

**Affiliations:** ^1^ Institut für Physikalische Chemie Heinrich‐Heine‐Universität Düsseldorf Düsseldorf Germany; ^2^ Fakultät für Chemie und Chemische Biologie Technische Universität Dortmund Dortmund Germany; ^3^ Institut für Organische Chemie und Makromolekulare Chemie Heinrich‐Heine‐Universität Düsseldorf Düsseldorf Germany

**Keywords:** charge transfer excitation, NIR spectroscopy, solvatochromism, thermally activated delayed fluorescence, time‐resolved spectroscopy

## Abstract

A thermally activated delayed fluorescence (TADF) emitter consisting of a triarylamine (TAA) donor and a 1,4‐dicyanobenzene (DCN) acceptor moiety was characterized by femtosecond UV‐Vis and near infrared (NIR) spectroscopy. The combination of the two techniques allows to probe spectral changes of the emitter in a range extending from 350 to 1600 nm. With the approach, low‐lying higher singlet excitations (S_n≥2_) contributing to intersystem crossing via spin vibronic mechanisms can be located energetically. Due to the charge transfer (CT) character of the S_1_ state, the transition energies S_1_→S_n≥2_ are strongly solvent dependent as experiments on TAA‐DCN dissolved in cyclohexane, toluene, 1,2‐dimethoxyethane, and acetonitrile indicate. The experiments also hold information on dielectric and vibrational relaxation ensuing S_1_ excitation.

## Graphical abstract

The depicted thermally activated delayed fluorescence (TADF) emitter was studied by femtosecond near‐infrared (NIR) spectroscopy. A prominent NIR transition was shown to be strongly solvent‐dependent, with implication for intersystem crossing in the compound.

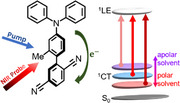

## 1 Introduction

In organic light‐emitting diodes (OLEDs), electron‐hole recombinations result in singlet (S_1,2,…_) and triplet (T_1,2,…_) excitations of molecular emitters [1, 2]. Ideally, the decay of both types of excitation results in light generation with quantum yields of one. Triplet states of purely organic emitters are typically “dark”, that is, they exhibit small radiative rate constants *k*_rad_ (typically in the range of 10^−1^ to 10^4^ s^−1^ compared to 10^8^ s^−1^ for singlet excitations) [3]. Thus, in organic emitters, other channels need to be operative to make use of the triplet excitations. In emitters utilizing thermally activated delayed fluorescence (TADF, also known as E‐type delayed fluorescence) [4, 5, 6, 7, 8], reverse intersystem crossing (rISC) from the T_1_ to the S_1_ state occurs. This is followed by the radiative decay of the S_1_ state. TADF requires small S_1_‐T_1_ gaps ($\Delta E_{\mathrm{ST}}$) of the order of the thermal energy $k_{B}T$ (∼ 25 meV at room temperature) [4, 9]. Such small energy gaps can be found in excitations with spatially separated electron and hole densities [10]. Charge transfer (CT) excitations of donor‐acceptor conjugates often feature small gaps. In the CT state, the hole is localized on the donor moiety and the electron on the acceptor part of the conjugate [11, 12, 13]. In multi‐resonance (MR) emitters hole and electron densities reside on adjacent atoms [14, 15, 16].

For either type of TADF emitter to operate, in addition to the thermodynamic condition (small $\Delta E_{\mathrm{ST}}$) also a kinetic one must be fulfilled. The equilibration of the S_1_ and T_1_ state (*k*_ISC_ *+k*_rISC_) needs to be substantially faster than the intrinsic decay of the T_1_ state (*k*_T_), *k*_ISC_ *+k*_rISC_ *> k*_T_. Otherwise, the T_1_ state will mostly decay nonradiatively to the ground state S_0_. For “pure” CT states, the rate constants *k*_ISC_ and *k*_rISC_ are usually small due to (nearly) vanishing spin‐orbit couplings (SOCs) [17, 18, 19]. Admixtures of states with local excitation character (LE) by vibronic and spin‐vibronic effects can enhance SOC and thereby enable the fast equilibration [19, 20, 21, 22]. Such admixtures imply that the S_1_ and T_1_ states acquire partial LE character, albeit they are nominally CT states. Importantly, this admixture does not correspond to a population of the LE states themselves. The Boltzmann distribution, together with energy gaps of around 10,000 cm^−1^ between CT and LE states prohibits the LE state population. Nevertheless, the energy gap remains highly relevant for ISC and rISC, as it appears in the pertinent expressions as an energy denominator for the ISC and rISC rate constants [23].

**FIGURE 1:**
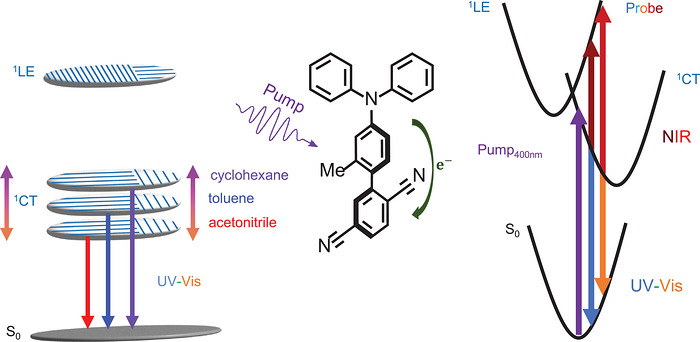
Energy‐level diagram depicting the low‐lying singlet states (^1^CT and ^1^LE) of the emitter (TAA‐DCN, shown in center) investigated by steady state methods (on the left side) and femtosecond transient absorption (on the right side). The low‐lying S_1_→S_n≥2_ transitions are expected to be in the NIR range.

In the spectroscopic characterization of TADF emitters, focus is usually laid on the S_1_ and T_1_ states and not on the LE states which are commonly higher excitations (S_n≥2_ and T_n≥2_). As shown recently by us [24], LE and other higher excited states can be addressed by time‐resolved near infrared (NIR) spectroscopy. The experiments were conducted on the CT‐type TADF emitter, 4′‐(diphenylamino)‐2′‐methyl‐[1,1′‐biphenyl]‐2,5‐dicarbonitrile (TAA‐DCN), consisting of a triarylamine (TAA) donor moiety and 1,4‐dicyanobenzene (DCN) acceptor part (for structure see Figure 1) dissolved in toluene. NIR characterization of its S_1_ state proved challenging as the instrument employed featured a response time of the same magnitude as the S_1_ lifetime (∼ 20 ns [25]). Meanwhile, we have constructed a femtosecond transient absorption (fsTA) NIR instrument with which S_1_→S_n≥2_ transitions can conveniently be addressed. With the instrument, the solvent impact on the NIR transition will be characterized and compared with expectations based on steady state spectroscopy. As CT‐excitations strongly respond to the solvent polarity [26, 27, 28, 29] (see Figure 1) pronounced effects are expected.

## 2 Results and Discussion

As this study aims to provide thorough insights into the S_1_→S_n≥2_ (especially the CT→LE) transition energies, and the CT excited states are strongly solvent‐dependent, solvents of different polarity were used. Cyclohexane (Cy, relative permittivity ε_r_ = 2.02 [30, 31]), toluene (Tol, ε_r_ = 2.38 [30, 31]), 1,2‐dimethoxyethane (DME, ε_r_ = 7.20 [30, 31]), acetonitrile (MeCN, ε_r_ = 35.94 [30, 31]), and dimethyl sulfoxide (DMSO, ε_r_ = 46.45 [30¸ 31]) were chosen as representative ones. Toluene was included to connect with our earlier study [24].

### 2.1 Steady State Spectroscopy

**FIGURE 2:**
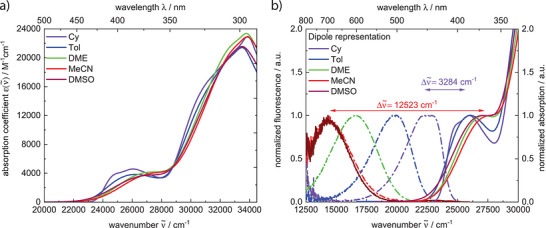
Steady state spectroscopy of TAA‐DCN in various solvents: (a) absorption coefficient spectra in cyclohexane (Cy, purple), toluene (Tol, blue), 1,2‐dimethoxyethane (DME, green), acetonitrile (MeCN, red), and dimethyl sulfoxide (DMSO, dark red). (b) Normalized absorption (solid lines) and fluorescence spectra (dashed lines) in the different solvents in the transition dipole representation. Stokes shifts ($\tilde{\upsilon }$_Stokes_) for cyclohexane (purple, smallest) and acetonitrile (red, largest) are indicated.

For all solvents considered, TAA‐DCN features an absorption band lowest in energy around 380 nm (Figure 2 and Table 1). Peak absorption coefficients in this range amount to ≈ 5000 M^−1^cm^−1^. In cyclohexane, different from the other solvents, a vibronic progression with one peak (around 380 nm) and a shoulder (around 406 nm) is observed. A weak hypsochromic shift (negative solvatochromic shift) is observed, that is the peaks are slightly blue shifted in polar solvents.

**TABLE 1:** Photophysical properties of TAA‐DCN from steady state absorption and fluorescence experiments. Absorption maxima λ_abs_ and absorption coefficients ε_max_ of the absorption bands lowest in energy are listed as well as emission maxima λ_em_. The 0‐0 energy ($E_{00}$), Stokes shift ($\Delta \tilde{\upsilon }$_Stokes_), vertical absorption, and emission energies were determined from absorption and fluorescence spectra converted into the transition dipole representation. The $E_{00}$ energies were obtained from three different approaches (see Experimental Section and Figure S1), and an average value was computed and is shown here as $\left\langle E_{00}\right\rangle$.

	Cy	Tol	DME	MeCN	DMSO
ε_r_	2.02^a^	2.38^a^	7.20^a^	35.94^a^	46.45^a^
λ_abs_ / nm	380	383	370	364	370
ε_max_ / M^−1^cm^−1^	4530	3840	4130	4040	3720
λ_em_ / nm	434	493	574	651	658
$\left\langle E_{00}\right\rangle$ / cm^−1^ (eV)	24040 ± 80 (3.0)	22640 ± 30 (2.8)	21520 ± 70 (2.7)	20850 ± 170 (2.6)	20320 ± 130 (2.5)
$\Delta \tilde{\upsilon }$_Stokes_ / cm^−1^	3284	6040	10032	12523	12206

^a^ Relative permittivities ε_r_ at 25°C reported here were obtained from ref. [30, 31].

Contrary to the absorption behavior, the fluorescence is strongly affected by the solvent environment. In cyclohexane the emission is centred around 434 nm, and in dimethyl sulfoxide, the solvent with the highest polarity in our study, the emission peaks at 658 nm (for the other solvents see Table 1). Vertical absorption and emission energies, Stokes shift, and 0‐0 energy ($E_{00}$) were retrieved from the absorption and emission spectra in the transition dipole representation (see Figure 2b)). 0‐0 energies ($E_{00}$) were computed based on the intersection of absorption and emission spectra, the average of the respective vertical energies, and the onsets (for details see Support Information Table S1 and Figure S1). The values obtained by these approaches are identical within ∼300 cm^−1^. An average value of the different approaches and the estimated error were computed. In cyclohexane, the largest 0‐0 energy ($\left\langle E_{00}\right\rangle$ amounts to 24040 cm^−1^) and the smallest Stokes shift (3284 cm^−1^) is observed. The smallest 0‐0 energy ($\left\langle E_{00}\right\rangle$ amounts to 20320 cm^−1^) and nearly the largest Stokes shift (12206 cm^−1^) are recorded in dimethyl sulfoxide.

While the strong positive solvatochromism of the emission is expected for a chromophore with a CT excitation [32, 33, 34], the (weak) negative solvatochromism of the absorption is puzzling at first sight. The increase of the absorption energy with increasing solvent polarity cannot be explained within the Onsager–Lippert–Mataga model [35, 36], since the model implies that a blue shift corresponds to either an opposite orientation of the dipole moments or that the dipole moment of the excited state ($|{\vec{\mu }}_{E}|$) is smaller than the dipole moment of the ground state ($|{\vec{\mu }}_{G}|$). As expected for a CT excitation, in a quantum chemical calculation on TAA‐DCN (see ref. [37], referred to as emitter 2‐Me) a strong increase of the dipole moment upon excitation to the S_1_ state (CT character) was computed. Kapturkiewicz et al. [38] observed the same behavior for related CT chromophores. They attributed the effect to the dipole moment of the molecule placed off‐center of the solvent cavity. The resulting arrangement of solvent dipoles in the ground state, creates a local field that stabilizes the ground state more strongly than the excited state. Immediately after photoexcitation, before any reorganization of the solvent can take place, this pre‐arrangement of the solvent field transiently destabilizes the excited charge‐transfer (CT) state. Consequently, a blue shift in the absorption is observed.

### 2.2 Transient Absorption Spectroscopy

**FIGURE 3:**
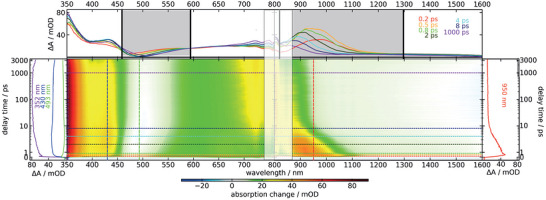
Transient absorption of TAA‐DCN in toluene in the UV‐Vis (left) and NIR (right) spectral region in the time range of 0 to 3.3 ns after excitation at 400 nm. In the contour plots, white to red colors represent positive difference absorptions. Vertical colored lines represent different time traces related to marked spectral positions and are plotted on the left (for UV‐Vis range) or on the right (for NIR range) of the contour plots. Horizontal lines represent different transient spectra related to indicated delay times and are plotted on top of the contour plots. The regions between the black vertical lines on top represent the spectral regions selected for the analysis of early time behavior. The white box marks the wavelength range where the data are not reliable due to the strong fundamental at 800 nm, which introduces artefacts and prevents trustworthy signal interpretation.

The S_1_→S_n_ transitions of TAA‐DCN in different solvents were accessed by fsTA in the UV‐Vis and NIR region. By combining results for both fsTA spectral regions, spectra covering the range of 350 – 1600 nm (with a small gap between 750 and 850 nm) and for delay times up to 3.4 ns were recorded. The description of the results focuses on TAA‐DCN in toluene (see Figure 3) as it features a “rich” behavior in the NIR.

**FIGURE 4:**
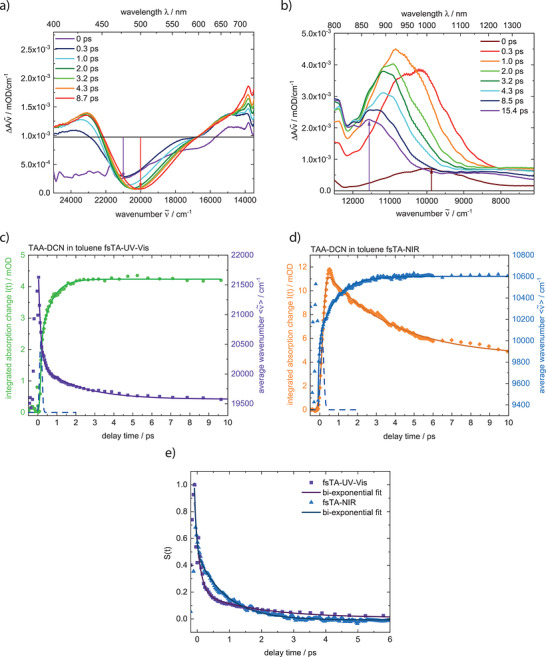
Comparison of spectral shifts of the transient absorption during the first picoseconds observed in the UV‐Vis (a) and NIR (b) range of TAA‐DCN in toluene. Analysis of the spectral shifts: Average wavenumbers (see Equation (3)) and band integrals (see Equation (4)) as a function of time are depicted in in the UV‐Vis (c) and NIR (d). The green and orange solid lines represent the results of single‐exponential fits which include an offset and take the instrument response function (IRF) (dashed‐blue lines) into account. Purple and blue solid lines represent bi‐exponential fits. Spectral response functions (average wavenumbers normalized) are compared in (e) (see. Equation (5)).

For all detection wavelengths and delay times, only positive difference absorption is observed. Negative signals due to ground state bleach (GSB) and stimulated emission (SE) cannot be observed directly. As detailed in ref. [24], the minimum around 500 nm is due to SE superimposing onto a stronger, positive contribution. On the picosecond time scale, the SE contribution shifts to larger wavelengths (dynamic Stokes shift, see Figure 4a)). In the NIR, a complementary behavior is observed (see Figure 4b)). Immediately after excitation, an absorption band with a maximum around 980 nm is observed, which shifts to shorter wavelengths and decreases in signal strength within a few picoseconds. For a quantitative comparison, time dependent average wavenumber $\left\langle \tilde{\nu }\right\rangle \left(t\right)$ and integrals I(t) were computed (for details see Experimental Section, Equations (3) and (4)).

The average emission wavenumber related to SE contributions immediately after excitation $\left\langle \tilde{\nu }\right\rangle \left(0\right)$ amounts to 21630 cm^−1^, and shifts to 19550 cm^−1^ for “infinite” times (same values as reported earlier [24]). The time‐dependent average wavenumbers were fitted with a bi‐exponential function (purple solid line in Figure 4c)), with time constants of 0.13 and 2.07 ps. The amplitude averaged decay time amounts to 0.57 ps. The absorption band in the NIR shows an average wavenumber of 9440 cm^−1^ immediately after excitation and shifts to 10590 cm^−1^ at longer delay times. A bi‐exponential function (blue solid line in Figure 4d)), accounts for this shift with time constants of 0.05 and 0.9 ps with an average decay time of 0.78 ps. To better quantify the differences between the average shifts in the UV‐Vis and NIR regions, the spectral response functions S(t)(see Experimental Section, Equation (5)) were calculated (see Figure 4e)). The comparison indicates a slightly slower shift of the NIR band in comparison to the shift of the SE contribution. The values observed in the UV‐Vis range are comparable to studies on dielectric relaxation in toluene [39, 40]. The integrals I(t) were fitted with single‐exponential functions including an offset and taking the IRFs into account (green and orange solid lines in Figure 4c) and d), respectively) yielding a time constant $\tau_{SE_{1}}=$0.6 ps (relative amplitude ‐1.9) and an offset of 4.2 for the UV‐Vis range. For the NIR, a time constant of $\tau_{\mathrm{ES}A_{1}}=$3.6 ps (relative amplitude 7.4) and an offset of 4.5 were determined. The temporal behaviors in the two regions are, thus, notably different.

With reference to Figure 1, one would expect the stimulated emission and NIR excited state absorption (ESA) to yield identical spectral response functions *S(t)*. Concerning the band integrals, identical time constants with amplitudes varying for the two ranges are expected. The pronounced differences in temporal behavior for the two probing ranges are intriguing. It might be related to the coexistence of two rotamers of TAA‐DCN [24]. These rotamers are nearly isoenergetic and therefore coexist in similar concentrations. One of these rotamers might dominate the SE contribution and the other one the NIR ESA. As in principle, the rotamers could differ in relaxation behavior, this might explain the differences observed.

**FIGURE 5:**
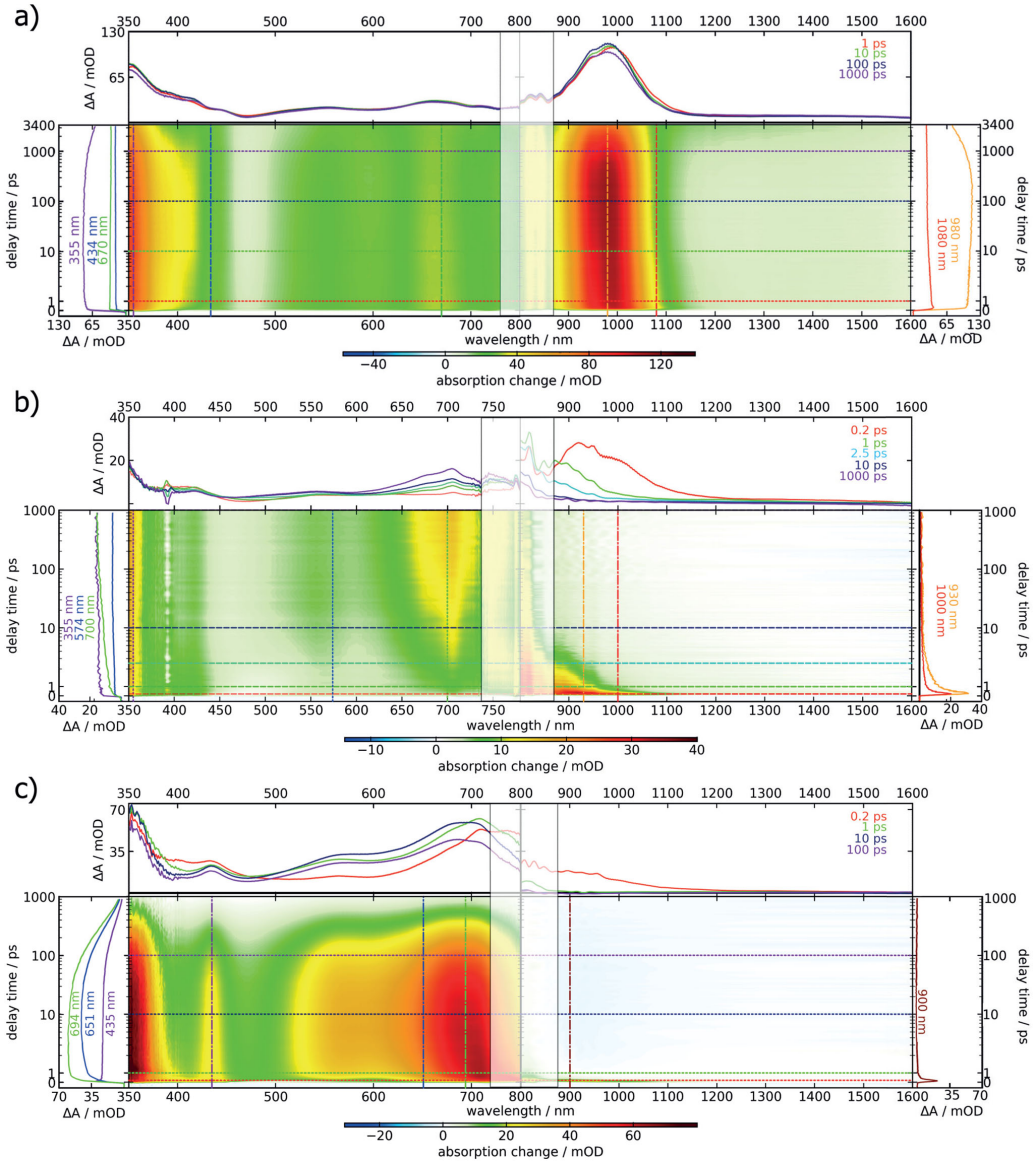
Transient absorption of TAA‐DCN in cyclohexane (a), 1,2‐dimethoxyethane (b), and acetonitrile (c) in the UV‐Vis (left) and NIR (right) spectral region in the time range of 0 to 3.3 ns after excitation at 400 nm. Vertical colored lines represent different time traces related to marked spectral positions and are plotted on the left (for UV‐Vis range) or on the right (for NIR range) of the contour plots. Horizontal lines represent different transient spectra related to indicated delay times and are plotted on top of the contour plots.

Similar to toluene, after photoexcitation strong ESA is observed in cyclohexane (Figure 5a)), 1,2‐dimethoxyethane (Figure 5b)), and acetonitrile (Figure 5c)). Differences between solvents become clear when looking into the NIR spectral range and taking the temporal behavior into account. In cyclohexane, no strong evidence of spectral shifts related to solvent relaxation is observed. In 1,2‐dimethoxyethane, ESA peaks around 950 nm immediately after excitation. Within picoseconds a hypsochromic shift is observed and after 10 ps no transient absorption in the NIR is discernible. In acetonitrile similar, albeit faster, behavior is observed. Immediately after excitation, ESA peaking around 900 nm is manifested. Within a picosecond, its NIR part vanishes. For both solvents, the behavior can be attributed to the S_1_ relaxation, as already observed in toluene. The difference in temporal behavior—the shift is slower in 1,2‐dimethoxyethane—matches reports on the dielectric relaxation times. In acetonitrile times constants of 0.089 and 0.63 ps [41], and in 1,2‐dimethoxyethane/water mixtures, time constants in ps to sub‐ps (∼80 ps) [42] timescales were reported.

From ∼10 ps until the end of the time range covered (up to 3.4 ns), the transient absorption is essentially constant, except for acetonitrile. This implies that the S_1_ lifetime exceeds ∼3 ns in all solvents except for acetonitrile for which a global analysis yields a lifetime $\tau_{S_{1}}^{\mathrm{MeCN}}$ of 360 ps. As shown for toluene, the S_1_ decay goes along with the population of the triplet state with high yields on a timescale of ∼10 ns [25]. Most likely, this also applies to cyclohexane and 1,2‐dimethoxyethane. In acetonitrile, the decay of the S_1_ signatures is not accompanied by the rise of a T_1_ signature, the accelerated decay in this solvent is due to internal conversion (IC) to the ground state. In line with the energy gap law [8, 43], this process is faster in solvents with lower S_1_ energies (see Table 1).

**FIGURE 6:**
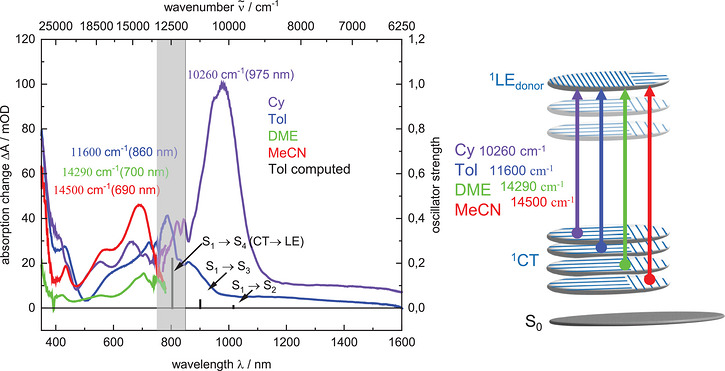
Transient spectra of TAA‐DCN obtained from fsTA‐UV‐Vis and/or fsTA‐NIR in cyclohexane at 1000 ps (purple line), toluene at 1000 ps (blue line), 1,2‐dimethoxyethane at 1000 ps (green line), and acetonitrile at 100 ps (red line). The low‐lying computed S_1_→S_n_ transitions of the compound in toluene are shown as black bars for comparison (values from ref. [24]).

In all solvents, from ∼10 ps onwards, the transient absorption represents the relaxed S_1_ state of TAA‐DCN. The experimental UV‐Vis to NIR S_1_→S_n≥2_ spectra obtained in the different solvents are plotted in Figure 6. According to the quantum chemical computations reported for TAA‐DCN in toluene [24], the vertical transition lowest in energy of the S_1_ state (S_1_→S_2_) is expected around 1000 nm. The predicted wavelength of the S_1_→S_3_ transition is 900 nm and for the S_1_→S_4_ 800 nm (see stick spectra in Figure 6). A comparison between the computed stick spectra with experimental spectra shows that with NIR probing one can address the lowest transition of the S_1_ state in this compound (see also Figure S2 in which a computed spectrum convoluted with Gaussian functions is shown). The lowest transition with pronounced CT→LE character is the S_1_→S_4_ transition which in toluene peaks at 860 nm. The local excitation is centered on the donor (TAA) moiety of the molecule (see Figure S3a)). In cyclohexane, this transition is bathochromically shifted to 975 nm. In the polar solvents, 1,2‐dimethoxyethane (700 nm) and acetonitrile (690 nm), a hypsochromic shift is observed.

**FIGURE 7:**
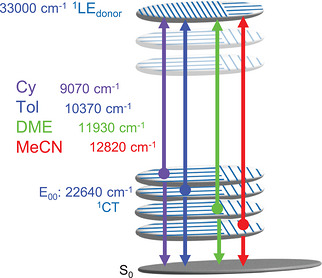
Prediction of S_1_→S_4_ (lowest CT→LE) transition of TAA‐DCN in toluene (blue), cyclohexane (purple), 1,2‐dimethoxyethane (green), and acetonitrile (red) based on the steady state absorption and fluorescence measurements. The 0‐0 energy ($\left\langle E_{00}\right\rangle$) and the vertical energy of the LE state in toluene obtained from the steady state spectroscopy are indicated.

The increase of CT→LE transition energies with solvent polarity reflects the decrease of the CT energy level with the polarity (see Figure 1). In the NIR experiment, the transition energies are vertical energies with respect to the S_1_ geometry. Such transition energies cannot be addressed by steady state spectroscopy. However, these transition energies may be estimated using vertical and 0‐0 energies. The vertical excitation energy (with respect to the S_0_ geometry) of the LE state was approximated by the excitation energy of TAA (see Figure S3). This energy is nearly solvent independent (Figure S4), with values ranging from 33,000 cm^−1^ (in toluene) to 33,670 cm^−1^ (in acetonitrile). By subtracting the 0‐0 energies of TAA‐DCN (see Table 1) from this vertical excitation energy of LE in the different solvents, approximate values for the CT→LE transition energies are obtained (Figure 7).

The estimated transition energies are of the right magnitude and obey the same solvent trends as the measured ones. Yet, the estimated values are by 1000–2500 cm^−1^ too small. The difference between estimated and measured transition energies is largest for TAA‐DCN in polar solvents. This is due to higher reorganization energies in these solvents.

## 3 Conclusion

This study has shown that with femtosecond NIR spectroscopy the lowest transitions (S_1_→S_n≥2_) of a TADF emitter promoted to its S_1_ state can be addressed. A transition assigned to the promotion of the emitter from the S_1_ state of CT character to a LE state shows the expected hypsochromic shift with solvent polarity. This transition energy increases with time. The temporal characteristics match the ones of dielectric relaxation in the respective solvents. From the fsTA spectra in the NIR region after dielectric relaxation, the energy gap between the ^1^CT and the lowest ^1^LE state can be extracted. Together with the respective gap in the triplet manifold (^3^CT→^3^LE), this gap is expected to affect ISC and rISC, as it appears in the relevant rate expressions through the energy denominator [23]. Identifying correlations between the ISC and rISC rate constants and these gaps is therefore subject of present studies. For processes in solution, the time dependence of CT→LE transition energy is not expected to be relevant, as ISC [27, 44] occurs on a much longer time scale than dielectric relaxations [41]. In solid matrices, however, this relaxation can be substantially slower [45] and therefore the dynamic shifts might impact the ISC kinetics.

## 4 Experimental Section

### 4.1 Sample and Common Conditions

**SCHEME 1:**
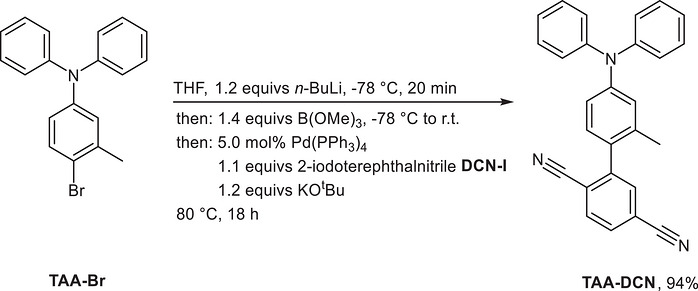
BLEBS sequence toward the targeted product TAA‐DCN.

The TADF compound, TAA‐DCN, was originally reported by G. A. Sommer et al. [25]. The synthesis was subsequently optimized using the BLEBS sequence [24, 46] (bromine‐lithium exchange‐borylation‐Suzuki), achieving a high yield by following the reported procedure [24] (Scheme 1). 4‐Bromo‐3‐methyl‐*N*,*N*‐diphenylaniline TAA‐Br (for synthesis, see Scheme S1) underwent a bromine‐lithium exchange at −78°C in THF to form the lithiated species, which was trapped with trimethyl borate to the corresponding boronate complex. Through a subsequent Suzuki cross‐coupling, this boronate complex was converted with 2‐iodoterephthalnitrile DCN‐I (for synthesis, see Scheme S2) in the presence of potassium *tert*‐butoxide and catalytic amounts of Pd(PPh_3_)_4_ at 80°C for 18 h to the target compound TAA‐DCN [24]. The ^1^H and ^13^C NMR spectra of the compounds are shown in Figures S5–S14.

The sample employed for the determination of the absorption coefficient and part of the time resolved measurements was synthesized with the method described above [24] (see Section S5 Supporting Information). Cyclohexane (≥99.7%, from Sigma–Aldrich), toluene (≥99.7%, from Sigma–Aldrich), 1,2‐dimethoxyethane (≥99.7%, from Sigma–Aldrich), acetonitrile (≥99.9%, from CHEMSOLUTE), and dimethyl sulfoxide (≥99.7%, from Sigma–Aldrich) were obtained commercially and used as received. All measurements were performed at room temperature (19°C–21°C).

### 4.2 Steady State Spectroscopy

A two‐beam UV‐Vis‐NIR spectrometer (Lambda 1050+ from PerkinElmer GmbH) was used to obtain the UV‐Vis absorption spectra with standard 0.1 cm path‐length fused silica cuvettes (from Hellma Analytics). A FluoroMax‐4 from Horiba Scientific was used to record emission spectra employing its right‐angle detection mode. To prevent inner filter effects, the absorption at the excitation wavelength was adjusted to ≤0.05 in a 1 cm cuvette. All emission spectra were corrected for the spectral sensitivity of the instrument, and for the Raman effect by subtracting a scaled spectrum of the solvent. The samples were excited at wavelengths close to their absorption maxima lowest in energy (∼380 nm).

### 4.3 Femtosecond Transient Absorption Spectroscopy

The femtosecond transient absorption (fsTA) setup which covers the UV‐Vis range was described elsewhere [47]. In the experiments reported here, the pulses (∼100 fs full width half maximum (FWHM), 800 nm, 1 kHz) of a Ti:Sa laser amplifier system (Libra from Coherent) were frequency doubled in a β‐barium borate crystal (BBO crystal type I, cut at θ=29.2°, 0.1 mm thick) to obtain the 400 nm pump pulses. The energy of the pump pulses was adjusted to around 1 µJ. The probe pulses were obtained by supercontinuum generation in a 5 mm thick CaF_2_ crystal. At the sample location, pump (diameter of about 160 µm (FWHM)) and probe (diameter of about 100 µm (FWHM)) were aligned with their relative polarization set to the magic angle (54.7°). Transient spectra were recorded equidistantly from ‐1 to 1 ps with respect to time zero on a linear and from 1 to 3.4 ns on a logarithmic time scale. For each delay setting, 2000 spectra were recorded, and the data were averaged over 4 successive delay‐line scans. The time resolution was about 150 fs (FWHM of the instrument response function (IRF)). A fused silica flow cell (custom made, Hellma Analytics) with 0.1 cm path length was used for circulating the sample solutions.

The fsTA‐NIR setup was recently implemented in our group and was not described heretofore. The same laser amplifier system as in the fsTA experiment with UV‐Vis probing provided the laser pulses. 400 nm pump pulses were also obtained using the same procedure and materials. The NIR probe light was generated by focusing the 800 nm pulses onto an 8 mm thick sapphire crystal (from Eksma Optics). As several studies have shown [48, 49, 50], for continuum generation in the NIR, crystal thicknesses should be larger than for UV‐Vis continuum. By carefully optimizing the focusing conditions (500 mm focal length, and f‐number of 60), a continuum spectrum extending to ∼1800 nm could be obtained (see Figure S15). After passing the sample, the NIR light is dispersed by a spectrograph (Acton Series SP‐2300i from Princeton Instruments) equipped with a plane ruled diffraction grating with 30 lines per mm and a nominal blaze at 1100 nm (from Newport). NIR radiation is detected by a 512 elements InGaAs photodiode array (Hamamatsu G11478‐512WB) included in a camera system (custom‐built to synchronize the detection system with two optical chopper systems and laser triggering from Entwicklungsbüro EB Stresing). The detection provides sensitivity up to ∼2500 nm. Contributions of second order diffraction to the signals recorded at longer wavelengths (∼1400‐1600 nm) were supressed by placing a long‐pass filter (20CGA‐1000, 1.1 mm thick, from Newport) in front of the respective pixels. The detection system is also sensitive in the Vis range (for wavelengths larger than ∼450 nm). Thus, transient spectra recorded with the NIR setup can overlap spectrally with the ones recorded by the UV‐Vis setup. This allows to scale signals recorded with the two instruments to identical excitation and probing conditions (see Figure S16). The time delay between pump and probe is controlled with a delay line (DL325, from Newport). With choppers placed in the pump as well as probe branch, the dark signal ($I_{\mathrm{bg}}\left(\lambda ,t\right)$), the probe spectrum ($I_{\mathrm{probe}}\left(\lambda ,t\right)$), pump contributions ($I_{\mathrm{pump}}\left(\lambda ,t\right)$), and the probe spectrum affected by the pump ($I_{\mathrm{pump}+\mathrm{probe}}\left(\lambda ,t\right)$) can be recorded. With these signals, the transient absorption (ΔA(λ,t)) was calculated using Equation (1): [51, 52]

(1) $$\Delta A\left(\lambda ,t\right)=-\log\frac{I_{\mathrm{pump}+\mathrm{probe}}\left(\lambda ,t\right)\;-\;I_{\mathrm{pump}}\left(\lambda ,t\right)}{I_{\mathrm{probe}}\left(\lambda ,t\right)\;-\;I_{\mathrm{bg}}\left(\lambda ,t\right)}.$$

The shift of time zero with the detection wavelength due to dispersion [53, 54] was calculated based on the group velocity dispersion (GVD) [55, 56] that the probe light experiences as it propagates through the optical components (such as the sapphire crystal, fused silica focusing lens, wired‐grid polarizer, etc) inserted in the path. The temporal shift was estimated and time zero was then corrected by subtracting it from the delay time. In addition, the temporal shift due to dispersion was also experimentally obtained using the optical Kerr effect [57] reaching a good agreement with the computed GVD values. This experiment also yielded the time resolution of the pump‐probe experiments which is ∼200 fs (FWHM of IRF). To assess the quality of the instrument, fsTA‐NIR measurements on β‐carotene in cyclohexane with the novel setup were compared with published ones [58, 59]. The spectral and temporal features were well reproduced (see Figure S17).

For the experiments presented here, pump (diameter of about 300 µm (FWHM)) and probe (diameter of about 240 µm (FWHM)) were focused onto the sample with the relative polarization set to magic angle (54.7°). The energy of the pump was adjusted to 1.75 µJ. Transient spectra, for the toluene solutions, were recorded equidistantly from −2 to 6 ps with respect to time zero (with 260 steps) and then until 40 ps (with 70 steps) and from 40 ps to 3.7 ns on a logarithmic time scale (with 150 steps). In cyclohexane, transient spectra were recorded equidistantly from −2 to 6 ps with respect to time zero (with 260 steps) and then until 30 ps (with 55 steps) and from 30 ps to 3.7 ns on a logarithmic time scale (with 150 steps). In acetonitrile, transient spectra were recorded equidistantly from −3 to 3 ps with respect to time zero (with 200 steps) and then until 30 ps (with 50 steps) and from 30 ps to 1.0 ns on a logarithmic time scale (with 40 steps). In 1,2‐dimethoxyethane, transient spectra were recorded equidistantly from −3 to 3 ps with respect to time zero (with 200 steps) and then until 30 ps (with 50 steps) and from 30 ps to 1.0 ns on a logarithmic time scale (with 40 steps). For all measurements, and for each delay setting 8000 spectra were averaged over one scan. A fused silica flow cell (Hellma Analytics) with 0.1 cm path length were used for circulating the sample solutions.

For both fsTA‐UV‐Vis and fsTA‐NIR experiments, the absorption of the sample solutions at 400 nm were adjusted to be 0.6‐0.7 per 0.1 cm. The transient absorption data were time zero corrected and solvent contributions were recorded in a separated experiment and subtracted with proper scaling [54]. The NIR spectra exhibit pixel‐to‐pixel variation due to the fixed pattern effect [60, 61]. For diminishing this effect, a Savitzky–Golary [62, 63] filtering was applied. At last, the fsTA‐NIR data was scaled to match the UV‐Vis signals as described above.

## 5 Data Analysis

To obtain the absorption maxima (λ_abs_) of the absorption coefficient band lowest in energy in the different solvents, the absorption coefficient spectra (from 600 to 300 nm) were decomposed in 3 Gaussian components (4 components for cyclohexane solutions), and the peak positions of the Gaussian function for the absorption lowest in energy extracted. To obtain the vertical excitation wavenumbers (${\tilde{\upsilon }}_{\mathrm{Abs},\max}$), vertical emission wavenumbers (${\tilde{\upsilon }}_{\mathrm{Fluor},\max}$), Stokes shifts ($\Delta \tilde{\upsilon }$_Stokes_), and the 0‐0 energies ($E_{00}$), fluorescence spectra were converted from constant wavelength (λ) to constant wavenumber ($\tilde{\upsilon }$) bandpass by multiplying them with $\lambda^{2}$ [64]. Then, the absorption coefficient spectra were rescaled according to ε($\tilde{\upsilon }$)/$\;\tilde{\upsilon }$, and fluorescence spectra according to F($\tilde{\upsilon }$)/$\;{\tilde{\upsilon }}^{3}$ to arrive at the transition dipole representation [65]. Finally, the normalized corrected absorption and fluorescence spectra were plotted and the $E_{00}$ energies were obtained from their intersection for cyclohexane and toluene solutions. For 1,2‐dimethoxyethane, acetonitrile, and dimethyl sulfoxide solutions the intersection approach was not suited for retrieving the $E_{00}$ energies, and the onsets of their absorption and emission spectra (see Figure S4 for example in toluene) and the vertical excitation and emission energies (using that $E_{00}$ = $({\tilde{\upsilon }}_{\mathrm{Abs},\max}+{\tilde{\upsilon }}_{\mathrm{Fluor},\max})/2$) were used as an alternative approach. The average values of the different approaches were computed to obtain the final $\left\langle E_{00}\right\rangle$ energies.

The transient absorption spectra (ΔA(λ,t)) obtained here are functions of the probe wavelength (λ) and the time delay (t) between pump and probe. To analyse these dependencies, two approaches were used. In the first one, time constants and decay‐associated difference spectra (DADS) were obtained using a global fitting procedure with a multi‐exponential trial function convoluted with the IRF (see Equation (2)) [66],

(2) $$\Delta A\;\left(\lambda ,t\right)=IRF⊗\sum_{i\;=1}^{n}\Delta A_{i}\left(\lambda \right)e^{-\frac{t}{\tau_{i}}}.$$

The IRF was approximated by a Gaussian function with a FWHM of 150 fs, and 200 fs for the fsTA‐UV‐Vis, and fsTA‐NIR data, respectively. The fit yields a decay‐associated difference spectrum (DADS) $\Delta A_{i}\left(\lambda \right)$ for each time constant $\tau_{i}$. In the second approach, only performed for toluene solutions, average wavenumbers ($\left\langle \tilde{\nu }\right\rangle \left(t\right)$, Equation (3)):

(3) $$\langle \tilde{\nu }\rangle \left(t\right)=\frac{\int_{{\tilde{\nu }}_{\min}}^{{\tilde{\nu }}_{\max}}\frac{\Delta A_{\mathrm{SE}}\left(\tilde{\nu },t\right)}{\tilde{\nu }}\tilde{\nu }d\tilde{\nu }}{\int_{{\tilde{\nu }}_{\min}}^{{\tilde{\nu }}_{\max}}\frac{\Delta A_{\mathrm{SE}}\left(\tilde{\nu },t\right)}{\tilde{\nu }}d\tilde{\nu }},$$

as well as spectral integrals (I(t), Equation (4)):

(4) $$I\;\left(t\right)=\int_{{\tilde{\nu }}_{\min}}^{{\tilde{\nu }}_{\max}}\frac{\Delta A_{\mathrm{SE}}\left(\tilde{\nu },t\right)}{\tilde{\nu }}d\tilde{\nu }$$

were computed for each delay time and these parameters were plotted as a function of the delay time. To this end, UV‐Vis transient absorption spectra $\Delta A\left(\tilde{\upsilon },t\right)/\tilde{\upsilon }$ were compared to the steady state fluorescence spectrum converted into the transition dipole representation (see ref. [24] for details). Then, the rescaled fluorescence was flipped and shifted [67], and the limits of the integrals (${\tilde{\nu }}_{\min}-{\tilde{\nu }}_{\max}$) used here were defined based on the spectral range in which both spectra overlap. For the NIR transient absorption spectra $\Delta A\left(\tilde{\upsilon },t\right)/\tilde{\upsilon }$, the limits of the integrals were defined as the spectral range of interest, from ${\tilde{\nu }}_{\min}\approx$7690 cm^−1^ (1/1300 nm^−1^) to ${\tilde{\nu }}_{\max}$ ≈12200 cm^−1^ (1/820 nm^−1^). Then the average wavenumber ($\left\langle \tilde{\nu }\right\rangle \left(t\right)$) from UV‐Vis and NIR were normalized to obtain the spectral response function S(t) [68, 69]:

(5) $$S\left(t\right)=\frac{\langle \tilde{\nu }\rangle \left(t\right)\;-\;{\langle \tilde{\nu }\rangle }_{\infty }}{{\langle \tilde{\nu }\rangle }_{0}\;-\;{\langle \tilde{\nu }\rangle }_{\infty }},$$

where ${\left\langle \tilde{\nu }\right\rangle }_{0}$ and ${\left\langle \tilde{\nu }\right\rangle }_{\infty }$ are the values of $\left\langle \tilde{\nu }\right\rangle \left(t\right)$ obtained at time zero and infinite time (for UV‐Vis that was around 24 ps and for NIR that was around 96 ps), respectively.

## Conflicts of Interest

The authors declare no conflicts of interest.

## Supporting information




**Supporting File 1**: Additional Supporting Information Can be Found Online in the Supporting Information section.

## Data Availability

The data that support the findings of this study are available from the corresponding author upon reasonable request.
