# Supplementary material for: Accessing Sn≥2 States of a TADF Emitter by Femtosecond NIR Spectroscopy
Source: Chemistry. 2026 Mar 23;32(26):e70875. doi: 10.1002/chem.70875 (PMC13356458; doi:10.1002/chem.70875)
Supplement: Supplementary file 1 — Supporting File 1: Additional Supporting Information Can be Found Online in the Supporting Information section. [file CHEM-32-e70875-s001.pdf]

Supporting Information

Accessing $S_{n\geq2}$ States of a TADF Emitter by Femtosecond NIR Spectroscopy

Wiebke Haselbach,[a] Jasmin Matthes,[a] Andreas Prüfer,[b] Simon L. Zimmermann,[a] Monika Flörke,[c] Thomas J. J. Müller,[c] Peter Gilch,*[a] Barbara E. Nogueira de Faria[a]

[a] W. Haselbach, J. Matthes, S. L. Zimmermann, Prof. Dr. P. Gilch, Dr. B. E. N. de Faria,
Institut für Physikalische Chemie
Heinrich-Heine-Universität Düsseldorf
Universitätsstraße 1, 40225 Düsseldorf, Germany

[b] A. Prüfer,
Fakultät für Chemie und Chemische Biologie
Technische Universität Dortmund
Otto-Hahn-Str. 6, 44227 Dortmund, Germany

[c] M. Flörke, Prof. Dr. Thomas J. J. Müller,
Institut für Organische Chemie und Makromolekulare Chemie
Heinrich-Heine-Universität Düsseldorf
Universitätsstraße 1, 40225 Düsseldorf, Germany

Correspondence: Peter Gilch (gilch@hhu.de), www.gilch.hhu.de

S1. Steady State Absorption and Emission

E_{00} Energy

To obtain the E_{00} energy of TAA-DCN in the different solvents, absorption coefficient as a function of wavenumber $\varepsilon(\tilde{\nu})$ were scaled according to $\varepsilon(\tilde{\nu})/\tilde{\nu}$, and fluorescence spectra $F(\tilde{\nu})$ according to $F(\tilde{\nu})/\tilde{\nu}^3$. Then the rescaled spectra were normalized. For acetonitrile and dimethyl sulfoxide solutions, the intersection between the rescaled absorption and emission spectra occurs at very low values or, in some cases, is absent. In these cases, the 0-0 energies were only determined by the average of the respective vertical energies $E_{00} = (\tilde{\nu}_{\mathrm{Abs,max}} + \tilde{\nu}_{\mathrm{Fluor,max}})/2$ and the "onset" approach (see Figure S1). As the values compiled in Table S1 show, the different approach yields consistent values.

Table S1. E_{00} energy in cm^{-1} obtained for TAA-DCN in the different solvents based on three different approaches: the onset, the average of the respective vertical energies, and the intersection.

E_{00} Approach	Cy	Tol	DME	MeCN	DMSO
Onset	23993	22651	21448	21014	20191
Average	24124	22604	21585	20681	20453
Intersection	23994	22650	21532	-	-

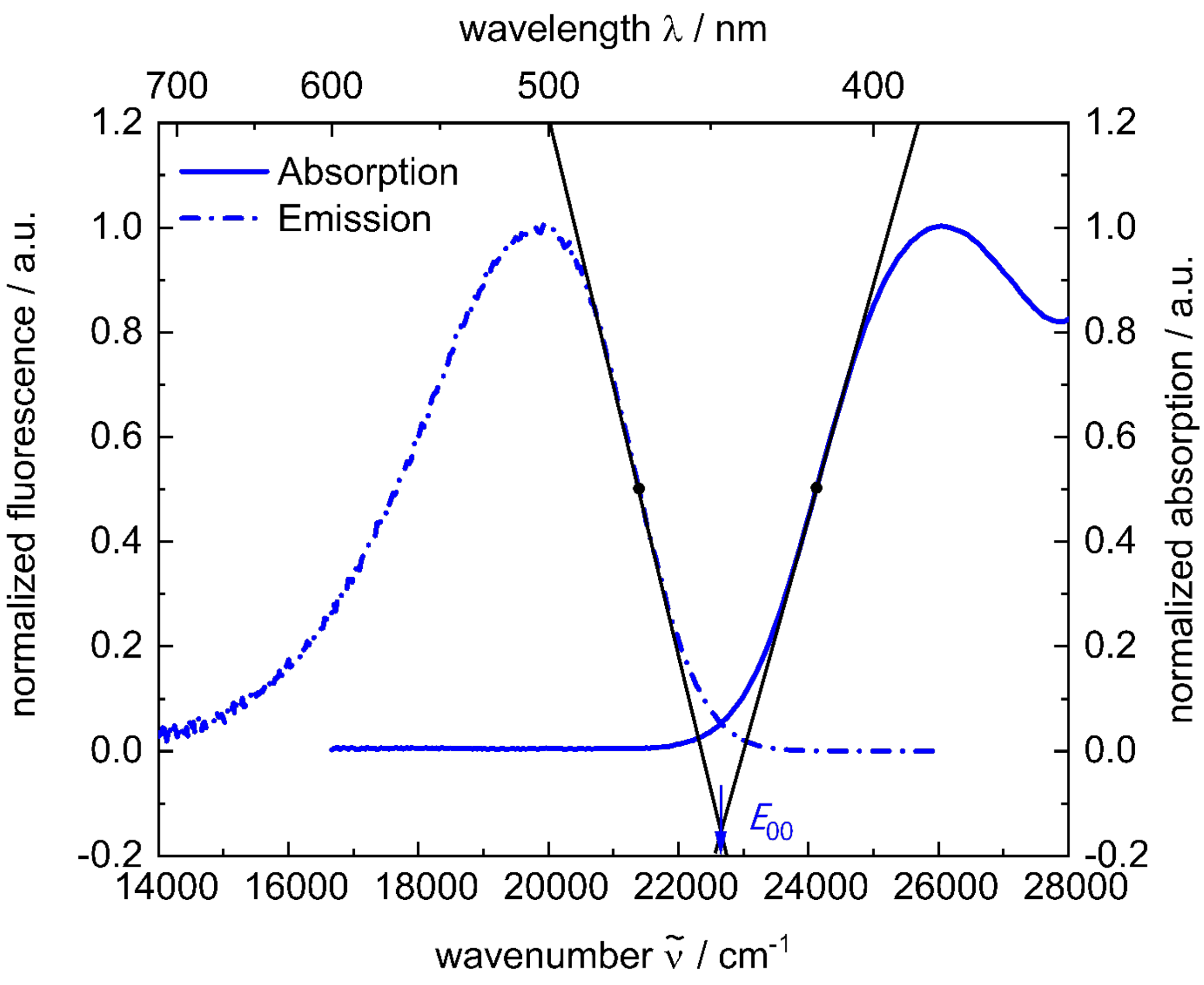

Figure S1. Onset approach to retrieve the E_{00} energy of TAA-DCN. Slopes at half maximum of the absorption and emission spectra were computed and the intersection of resulting lines determined. The E_{00} energy refers to the wavenumber of the point where the lines intersect.

S2. Lowest $S_1 \rightarrow S_n$ transitions of TAA-DCN in toluene determined by femtosecond and nanosecond NIR spectroscopy

The NIR signatures of the relaxed S_1 state of TAA-DCN in toluene recorded with the fs-NIR setup compare favourably with a quantum chemical prediction (Figure S2). To facilitate this prediction the computed stick spectra were convoluted with a Gaussian function. Note that the lowest three transitions $S_1 \rightarrow S_{2,3,4}$ when convoluted translate into a single broad Gaussian peak. The two spectra also match one recorded previously[1] with a nanosecond instrument (nsTA-NIR).

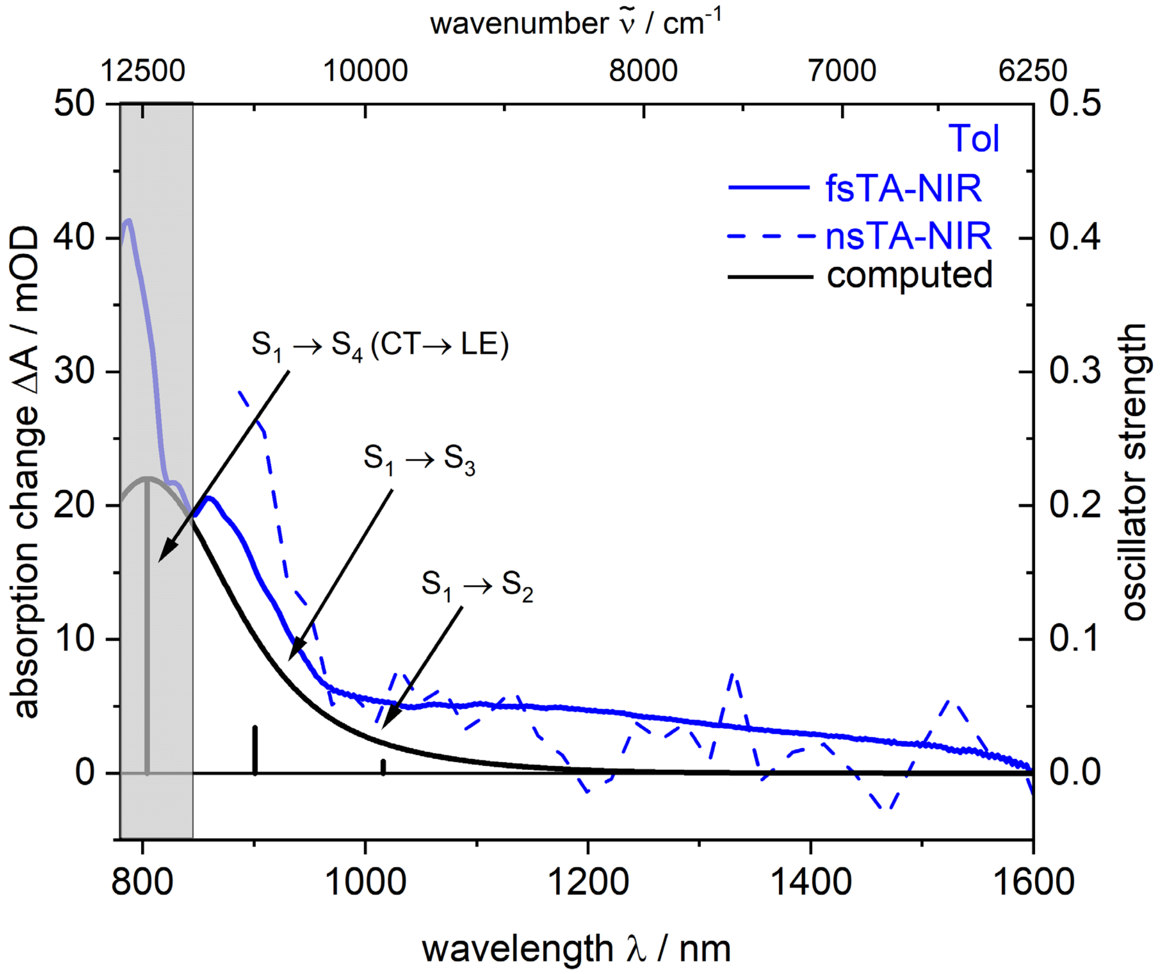

Figure S2. Comparison of the measured fsTA-NIR (blue solid line) and nsTA-NIR (blue dotted line) $S_1 \rightarrow S_{n\geq2}$ spectra with computed ones of TAA-DCN in toluene convoluted with a Gaussian function (black solid line).

S3. Decomposition of TAA-DCN Absorption into Donor, Acceptor and CT Contributions

The TAA-DCN emitter investigated here consists of a triarylamine (TAA) donor moiety and 1,4-dicyanobenzene (DCN) acceptor part. The absence of donor-only or acceptor-only absorption around 400 nm highlights the CT character of S_1 state (see Figure S3a)). Around 300 nm (33003 cm^{-1}) the absorption of TAA-DCN seems to be dominated by the local excitation of the donor moiety. Respective quantum chemical computations[1] are in line with this assignment. The computed transition energies are compared with an experimental spectrum in Figure S3b).

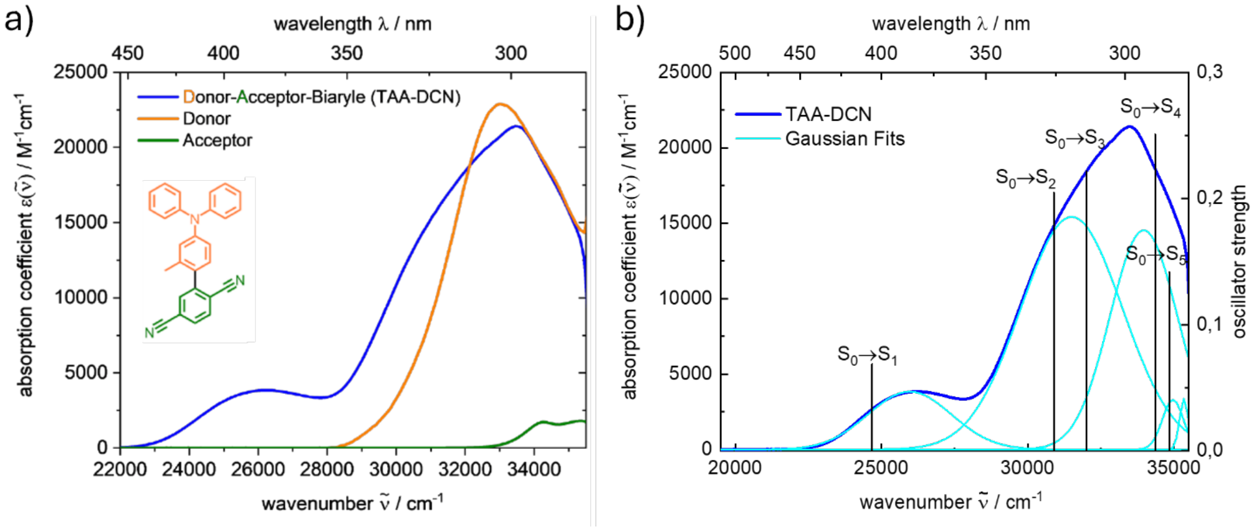

Figure S3. Absorption coefficient spectra in the UV-Vis: a) TAA-DCN emitter (in blue) with its donor (in orange) and acceptor (in green) moieties in toluene. b) Convoluted (blue) and Gaussian decomposition of TAA-DCN absorption spectrum (light blue solid lines) and the quantum chemical calculations of the lowest transitions from the S_0 state (black sticks).

S4. Excitation Energy of the Donor Moiety (TAA)

The normalized absorption spectra of the donor moiety (TAA) in the different solvents are shown in Figure S4. The vertical excitation energies (with respect to the S_0 geometry) of the LE state were approximated by the excitation energy of TAA (wavenumber peaks shown in Figure S4).

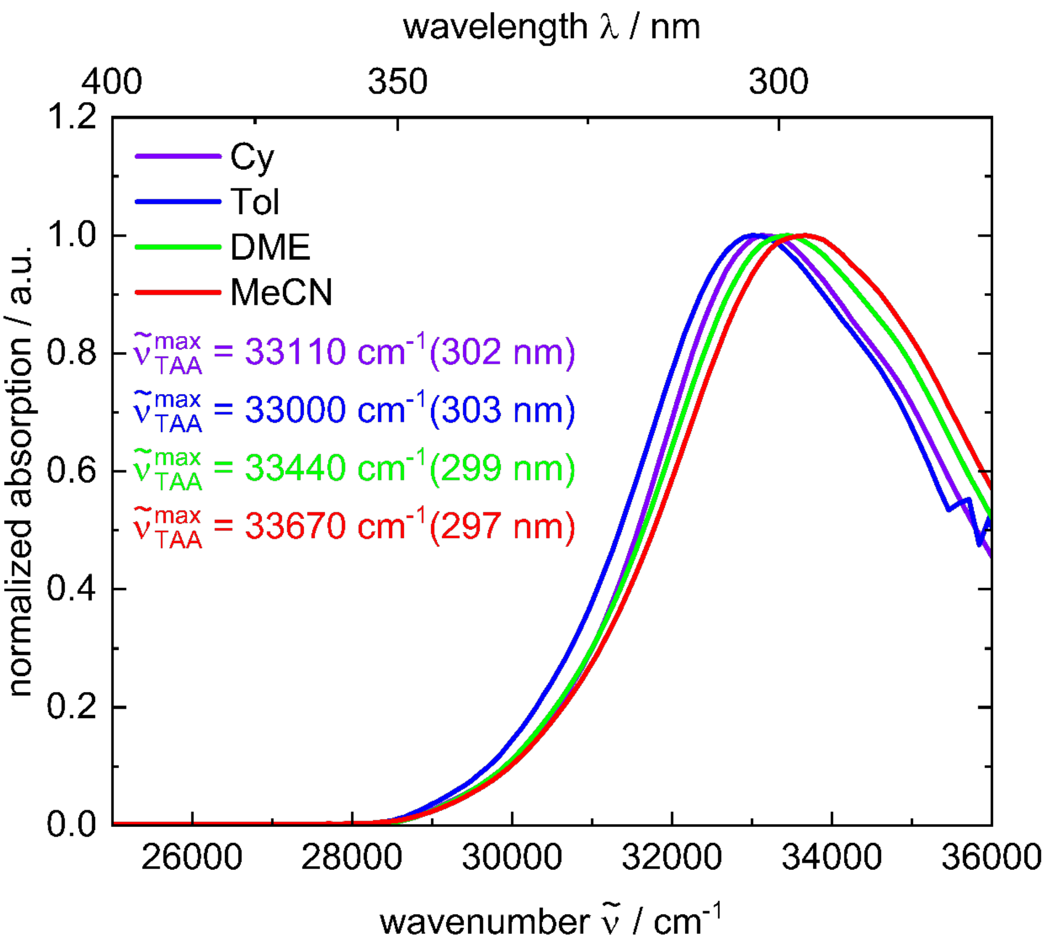

Figure S4. Normalized UV-Vis absorption spectrum of TAA in cyclohexane (purple), toluene (blue), 1,2-dimethoxyethane (green), and acetonitrile (red)

S5. Synthesis and Characterization

General considerations

All reactions were conducted in oven-dried glassware using standard Schlenk technique with septa and syringes under nitrogen atmosphere. The synthesis of DMT-I was carried out under air using a multi-neck flask. Reaction temperatures were controlled using preheated silicone oil or cooling baths (ice/water for 0 °C or dry ice/acetone for −78 °C). All compounds are known in the literature and were synthesized following previously reported procedures[1-2]. Reagents and catalysts were purchased reagent-grade from BLD Pharm, Acros Organics, Alfa Aesar, and VWR Chemicals and used without purification. THF was dried by a solvent purification system (MB-SPS 800 solvent drying system by M. Braun). Toluene was dried over activated 4 Å molecular sieve. The reaction progress was monitored qualitatively using TLC Silica gel 60 F234 aluminium sheets obtained from MACHEREY-NAGEL GmbH & Co. KG. The spots were detected with UV light at 254 and 365 nm. Purification of the compounds was performed on silica gel 60 M (0.04-0.063 mm) from MACHEREY-NAGEL GmbH & Co. KG using flash technique under pressure of 2 bar, unless otherwise indicated. Crude mixtures were absorbed onto Celite® 545 (Carl Roth GmbH & Co. KG) prior to chromatographic purification. As eluent distilled *n*-hexane and ethyl acetate were used. During recrystallization, heating was stopped, once the crude product was completely dissolved. By leaving the flask in the silicone oil bath, slow cooling of the solution and crystal formation was ensured. The isolated products were dried under vacuum (10^{-2} to 10^{-3} mbar) for at least 24 h.

The characterization was primarily conducted by the department CeMSA@HHU at Heinrich Heine University Düsseldorf. ^{1}H, ^{13}C and 135-DEPT ^{13}C NMR spectra were recorded on Bruker AVIII-300 and AVIII-600 spectrometers. DMSO-d_6 was used as deuterated solvent. The residual solvent signal was used as an internal standard (DMSO-d_6: ^{1}H δ 2.50, ^{13}C δ 39.52; water* in DMSO-d_6 ^{1}H δ 3.33). Signal multiplicities are abbreviated as follows: s: singlet; d: doublet; dd: doublet of doublet; ddd: doublet of doublet of doublets; m: multiplet. The type of carbon nucleus was determined based on 135-DEPT ^{13}C NMR spectra. For the ^{13}C NMR spectra assignments primary carbon nuclei are abbreviated with CH_3, secondary carbon nuclei with CH_2, tertiary carbon nuclei with CH and quaternary carbon nuclei with C_{quat}. EI mass spectra were recorded on Finnigan MAT TSQ 7000. Reported are all EI mass peaks with an intensity > 10% of the base peak and the mole peak. Melting points (uncorrected) were determined using a Büchi Melting Point B-540 instrument. Elemental analyses were performed with a PerkinElmer Series II Analyser 2400 at the microanalytical laboratory of the Institute for Pharmaceutical and Medical Chemistry at Heinrich Heine University.

* Due to the water content in DMSO-d_6, a signal for HDO is observed in the ^{1}H NMR spectra and labelled as "water".

Synthesis

The donor moiety, 4-bromo-3-methyl-*N*,*N*-diphenylaniline TAA-Br was synthesized via an Ullman coupling between iodobenzene and 4-bromo-3-methylaniline 3MA-Br using copper(I) iodide and 1,10-phenanthroline as the reactive catalyst/ligand system, under basic conditions with potassium *tert*-butoxid (Scheme S1).[2]

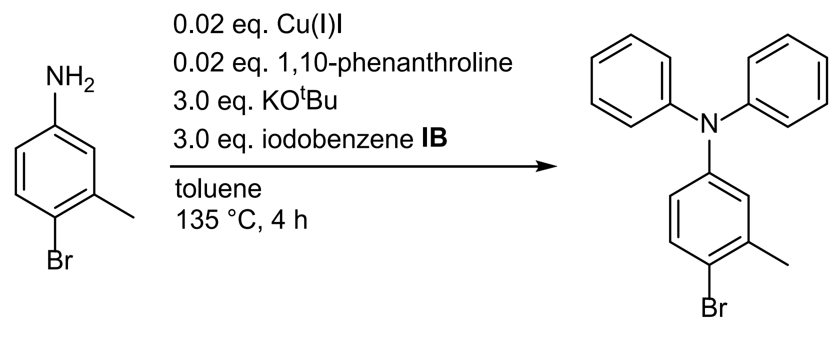

3MA-Br **TAA-Br**, 56%

Scheme S1. Ullmann coupling towards the donor moiety TAA-Br.

The acceptor moiety 2-iodoterephthalnitrile DCN-I was synthesized via a three-step sequence (Scheme S2). Starting from dimethyl-2-aminoterephthalate DMT-A, a Sandmeyer-type reaction was employed. A formation of the nitrosylium ion with sodium nitrite, was followed by an induced iodine exchange with potassium iodide resulted into 2-iodoterephthalate DMT-I. Afterwards the ester moieties of DMT-I were converted to amide functionalities by treating with aqueous ammonia solution in a Schlenk tube under reflux, yielding in 2-iodterephthalamide DAT-I. Finally, dried DAT-I was transformed into 2-iodoterephthalonitrile DCN-I via heating in phosphoryl chloride.[2]

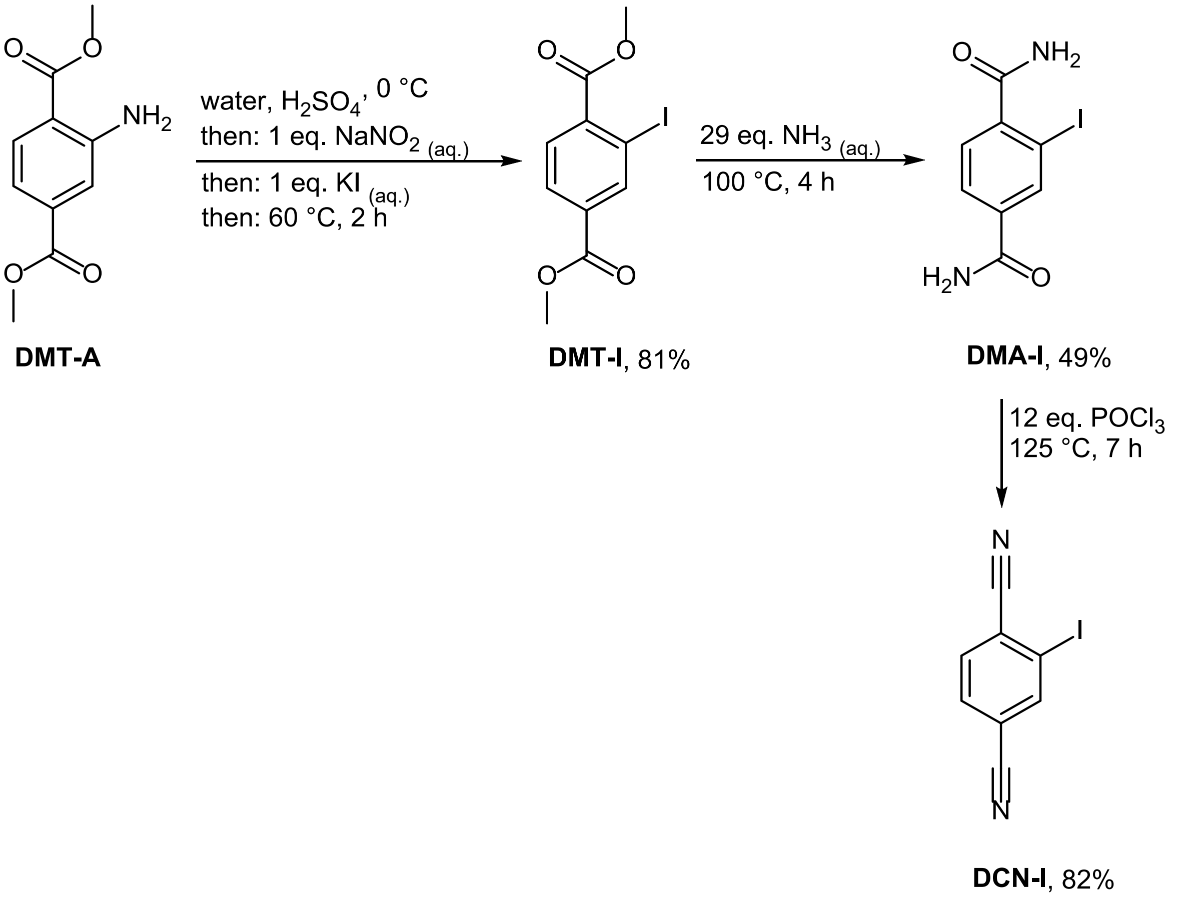

Scheme S2. Synthesis procedure towards the acceptor moiety DCN-I.

Synthesis conditions and characterization

Dimethyl-2-iodoterephthalate (DMT-I)[2]

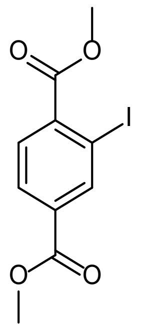

$C_{10}H_9IO_4$

[320.08]

In a two-necked flask equipped with a reflux condenser and dropping funnel, 5.25 g dimethyl-2-aminoterephthalate (1.00 equivs, 25.0 mmol) was suspended in 55.0 mL of distilled water under ice cooling and then mixed with 15.5 mL concentrated sulphuric acid. With continued cooling, an aqueous solution of 1.83 g sodium nitrite dissolved in 10.0 ml distilled water (1.00 equivs, 25.0 mmol) was added over a period of 30 min. Stirring was continued for

additional 30 min without cooling. Then, under renewed cooling, an aqueous solution of 4.98 g potassium iodide dissolved in 15.0 ml distilled water (1.20 equivs, 30.0 mmol) was added dropwise and stirred until gas evolution ceased. The resulting dark red suspension was heated to 60 °C and stirred for 2 h. After cooling to room temperature, the brown precipitate was filtered and washed with water. The crude product was diluted with ethyl acetate and purified by column chromatography on silica gel (*n*-hexane/ethyl acetate 10:1). The purified compound was obtained as a yellow solid with a yield of 6.48 g (20.2 mmol, 81%). Mp. 78 °C. R_f = 0.30 (*n*-hexane/ethyl acetate 10:1). ^{1}H NMR (600 MHz, DMSO-d$_6$) δ = 8.44 (d, ^4J = 1.6 Hz, 1H), 8.02 (dd, ^3J = 8.0 Hz, ^4J = 1.6 Hz, 1H), 7.80 (d, ^3J = 7.9 Hz, 1H), 3.89 (s, 3H), 3.88 (s, 3H). ^{13}C NMR (151 MHz, DMSO-d$_6$) δ = 166.6 (C_{quart}), 164.2 (C_{quart}), 140.5 (CH), 140.1 (C_{quart}), 132.9 (C_{quart}), 130.2 (CH), 128.7 (CH), 94.0 (C_{quart}), 52.7 (CH_3), 52.6 (CH_3). EI MS (70 eV, *m*/*z* (%)) 320 ([M]$^+$, 67), 289 ([$C_9H_6IO_3$]$^+$, 100), 290 ([$C_8H_3IO_4$]$^+$, 9), 261 ([$C_8H_6IO_2$]$^+$, 19), 246 ([$C_7H_3IO_2$]$^+$, 11), 75 ([C_6H_3]$^{3+}$, 9) (for ^{1}H and ^{13}C NMR spectra see Figure S5 and S6 respectively).

2-Iodoterephthalamide (DMA-I)[2]

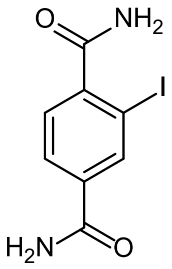

$C_{10}H_9IN_2O_2$

[290.06]

Under nitrogen in a dried Schlenk tube, 5.10 g dimethyl-2-iodoterephthalate DMT-I (1.00 equivs, 16.0 mmol) was suspended in 35.0 mL concentrated aqueous ammonia (28.9 equivs, 462 mmol) solution (25%) and then stirred at 100 °C for 4 h. After cooling to room temperature, the precipitated solid was filtered and washed with cold water. The purified compound was obtained as a colourless solid with a yield of 2.27 g (7.83 mmol, 49%). Mp. 91 °C. ^{1}H NMR (300 MHz, DMSO-d$_6$) δ = 8.32 (d, ^4J = 1.6 Hz, 1H), 8.11 (s, 1H), 7.92 – 7.85 (m, 2H), 7.60 (s, 1H), 7.52 (s, 1H), 7.40 (d, ^3J = 7.9 Hz, 1H). ^{13}C NMR (75 MHz, DMSO-d$_6$) δ = 170.3 (C_{quart}), 165.9 (C_{quart}), 145.5 (C_{quart}), 138.0 (CH), 135.8 (C_{quart}), 127.5 (CH), 127.0 (CH), 92.9 (C_{quart}). EI MS (70 eV, *m*/*z* (%)) 290 ([M]$^+$, 100), 274 ([$C_8H_5NIO_2$]$^+$, 80), 246 ([C_7H_5NOI]$^+$, 12), 229 (11), 163 ([$C_8H_7N_2O_2$]$^+$, 12), 119 (10), 119 (10), 75 ([C_6H_3]$^{3+}$, 8) (for ^{1}H and ^{13}C NMR spectra see Figure S7 and S8 respectively).

2-Iodoterephthalnitrile (DCN-I)[2]

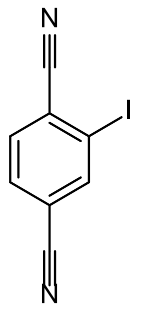

$C_8H_3IN_2$

[254.03]

Under nitrogen in a dried Schlenk tube, 0.57 g 2-iodoterephthalamide DMA-I (1.00 equivs, 1.90 mmol) was suspended in 2.00 mL phosphoryl chloride (11.6 equivs, 21.9 mmol) and stirred at 125 °C for 7 h. After cooling to room temperature, the excess of phosphoryl chloride in the reaction mixture was hydrolysed by addition of distilled water under ice cooling. The precipitated solid was filtered and washed with water. The purified compound was obtained as a grey solid with a yield of 0.40 g (1.57 mmol, 82%). Mp. 221 °C. ^{1}H NMR (300 MHz, DMSO-d$_6$) δ = 8.59 (d, ^4J = 1.4 Hz, ^5J = 0.6 Hz, 1H), 8.10 – 8.02 (m, 2H). ^{13}C NMR (75 MHz, DMSO-d$_6$) δ = 142.1 (CH), 135.0 (CH), 132.2 (CH), 123.5 (C_{quart}), 118.6 (C_{quart}), 116.5 (C_{quart}), 116.3 (C_{quart}), 101.0 (C_{quart}). EI MS (70 eV, *m*/*z* (%))

254 ([M]$^+$, 100), 128 (16), 127 ([$C_8H_3N_2$]$^+$, 59), 101 ([C_7H_3N]$^{2+}$, 5), 100 (19) (for ^{1}H and ^{13}C NMR spectra see Figure S9 and S10 respectively).

4-Bromo-3-methyl-N,N-diphenylaniline (TAA-Br)[2]

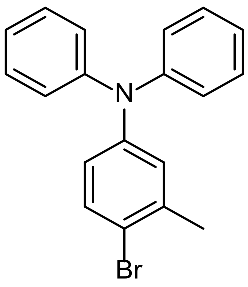

$C_{19}H_{16}BrN$

[338.25]

Under nitrogen in a dried Schlenk tube, 0.74 g 4-bromo-3-methylaniline 3MA-Br (1.00 equivs, 4.00 mmol), 0.02 g (0.02 equivs, 0.08 mmol) copper(I) iodide, 0.02 g (0.02 equivs, 0.08 mmol) 1,10-phenanthroline, and 1.30 g (3.00 equivs, 12.0 mmol) of potassium *tert*-butoxide were added and dissolved in 10.0 mL toluene. Then 1.32 mL iodobenzene IB (3.00 equivs, 12.0 mmol) was added and the reaction mixture was stirred at 135 °C for 4 h. After cooling to room temperature, the crude product was diluted with ethyl acetate and purified by column chromatography on silica gel (*n*-hexane/ethyl acetate 20:1). The purified compound was obtained as a brown solid with a yield of 0.75 g (2.24 mmol, 56%). Mp. 135 °C. R_f = 0.76 (*n*-hexane/ethyl acetate 20:1). ^{1}H NMR (600 MHz, DMSO-d_6) δ = 7.45 (d, ^3J = 8.6 Hz, 1H), 7.32 – 7.28 (m, 4H), 7.07 – 6.95 (m, 7H), 6.71 (dd, ^3J = 8.6 Hz, ^4J = 2.8 Hz, 1H), 2.23 (s, 3H). ^{13}C NMR (151 MHz, DMSO-d_6) δ = 146.9 (C_{quart}), 146.8 (C_{quart}), 138.2 (C_{quart}), 132.9 (CH), 129.6 (CH), 125.5 (CH), 124.0 (CH), 123.3 (CH), 122.7 (CH), 116.9 (C_{quart}), 22.5 (CH_3). EI MS (70 eV, *m*/*z* (%)) 340 (17), 339 ([^{81}Br-M]$^+$, 91), 338 (26), 337 ([^{79}Br-M]$^+$, 100), 259 (28), 258 ([$C_{19}H_{16}N$]$^+$, 32), 257 (67), 256 (30), 254 (13), 244 ([$C_{18}H_{14}N$]$^+$, 15), 243 ([$C_{18}H_{13}N$]$^{2+}$, 59), 242 (24), 241 (18), 181 ([$C_{13}H_{11}N$]$^+$, 23), 180 (67), 179 (14), 178 (16), 167 ([$C_{12}H_9N$]$^+$, 36), 166 (29), 165 (15), 155 (15), 154 (11), 153 (23), 152 (33), 151 (11), 141 (12), 140 (14), 139 (15), 129 (16), 128 (24), 127 (18), 120 (18), 115 (21), 91 (14), 90 ([C_6H_4N]$^+$,16), 89 (26), 78 (22), 77 ([C_6H_5]$^+$, 61), 63 (13), 51 (42) (for ^{1}H and ^{13}C NMR spectra see Figure S11 and S12 respectively).

4'-(Diphenylamino)-2'-methyl-[1,1'-biphenyl]-2,5-dicarbonitrile (TAA-DCN)[1]

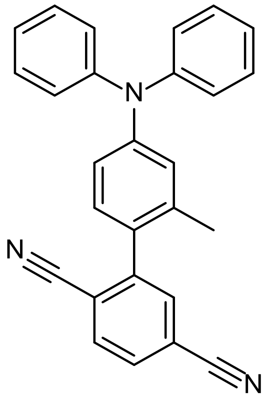

$C_{27}H_{19}N_3$

[385.47]

Under nitrogen in a dried Schlenk tube, 0.34 g (1.00 equivs, 1.00 mmol) of 4-bromo-3-methyl-*N*,*N*-diphenylaniline TAA-Br was dissolved in 5.00 mL THF and cooled to −78 °C. Then 0.82 mL (1.20 equivs, 1.20 mmol) *n*-butyllithium solution in hexanes (1.39 M) were added and the reaction mixture was stirred for 20 min. Afterwards, 0.16 mL (1.40 equivs, 1.40 mmol) of trimethyl borate were added at −78 °C and then warmed to room temperature. For the following Suzuki coupling 0.06 g (0.05 equivs, 0.05 mmol) of $Pd(PPh_3)_4$, 0.27 g (1.05 equivs, 1.05 mmol) of 2-iodoterephthalnitrile, and 0.13 g (1.20 equivs, 1.20 mmol) of potassium *tert*-butoxide were added before the reaction mixture was heated at 80 °C for 18 h. After cooling to room temperature, the crude product was diluted with ethyl acetate and purified by column chromatography on silica gel (*n*-hexane/ethyl acetate 5:1). After recrystallization

from ethanol, a yellow solid was obtained with a yield of 0.36 g (0.94 mmol, 94%). Mp. 164 °C. R_f = 0.37 (*n*-hexane/ethyl acetate 5:1). ^{1}H NMR (300 MHz, DMSO-d_6) δ = 8.15 (dd, ^3J = 7.7 Hz, ^4J = 1.0 Hz, 1H), 8.08 – 7.99 (m, 2H), 7.38 – 7.29 (m, 4H), 7.15 (d, ^3J = 8.3 Hz, 1H), 7.12 – 7.03 (m, 6H), 6.92 (d, ^4J = 2.3 Hz, 1H), 6.84 (dd, ^3J = 8.3, ^4J = 2.4 Hz, 1H), 2.02 (s, 3H). ^{13}C NMR (75 MHz, DMSO-d_6) δ = 148.2 (C_{quart}), 146.8 (C_{quart}), 145.5 (C_{quart}), 136.7 (C_{quart}), 134.3 (CH), 134.2 (CH), 131.6 (CH), 130.8 (CH), 129.8 (CH), 129.7 (C_{quart}), 124.8 (CH) , 123.8 (CH), 123.3 (CH), 119.5 (CH), 117.5 (C_{quart}), 117.1 (C_{quart}), 116.4 (C_{quart}), 115.6 (C_{quart}), 19.71 (CH_3). EI MS (70 eV, *m*/*z* (%)) 387 (30), 386 ([M]$^+$, 100), 385 (32), 167 (15), 166 (10), 77 ([C_6H_5]$^+$, 12). Anal. calcd. for $C_{27}H_{19}N_3$ (%) C 84.13, H 4.97, N 10.90; Found C 84.36, H 5.13, N 10.89 (for ^{1}H and ^{13}C NMR spectra see Figure S13 and S14 respectively).

^{1}H and ^{13}C NMR spectra

Dimethyl-2-iodoterephthalate (DMT-I)

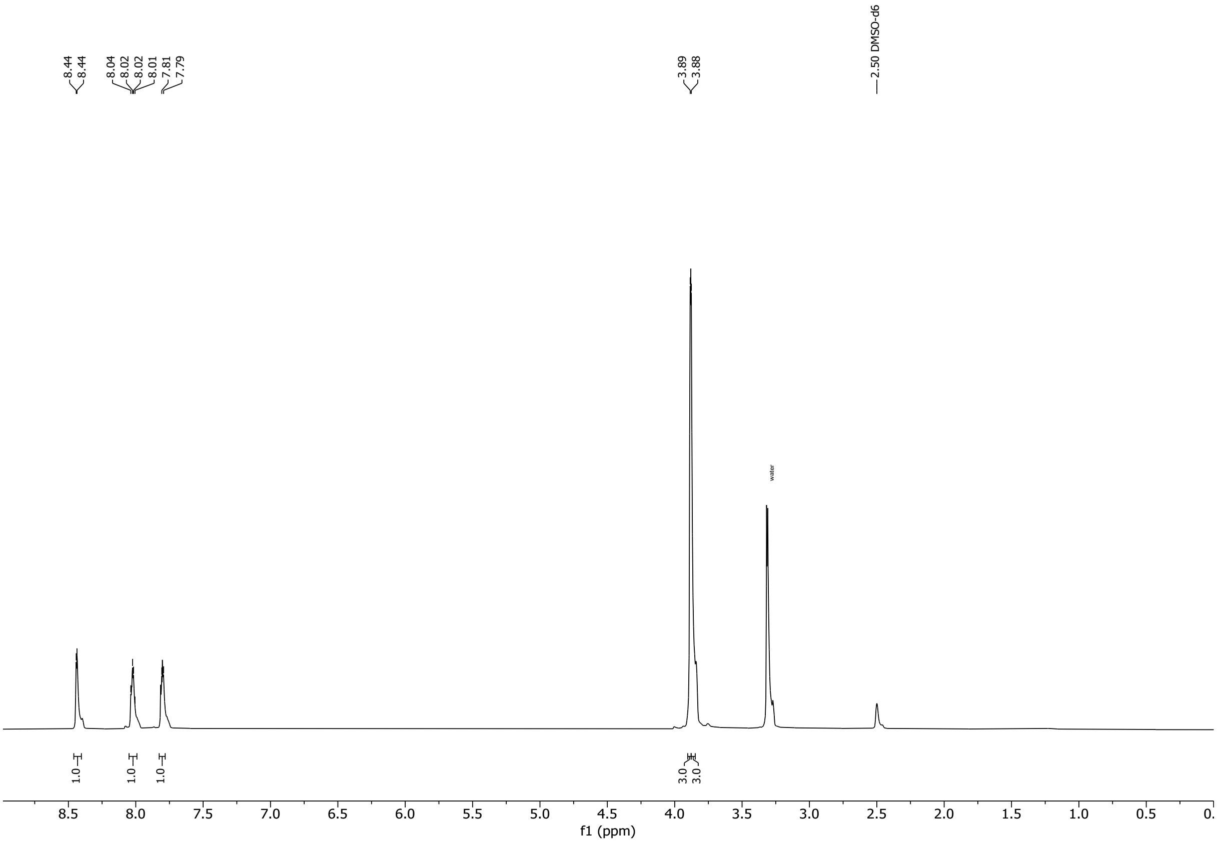

Figure S5. ^{1}H NMR spectrum of DMT-I (600 MHz, DMSO-d_6, 302 K).

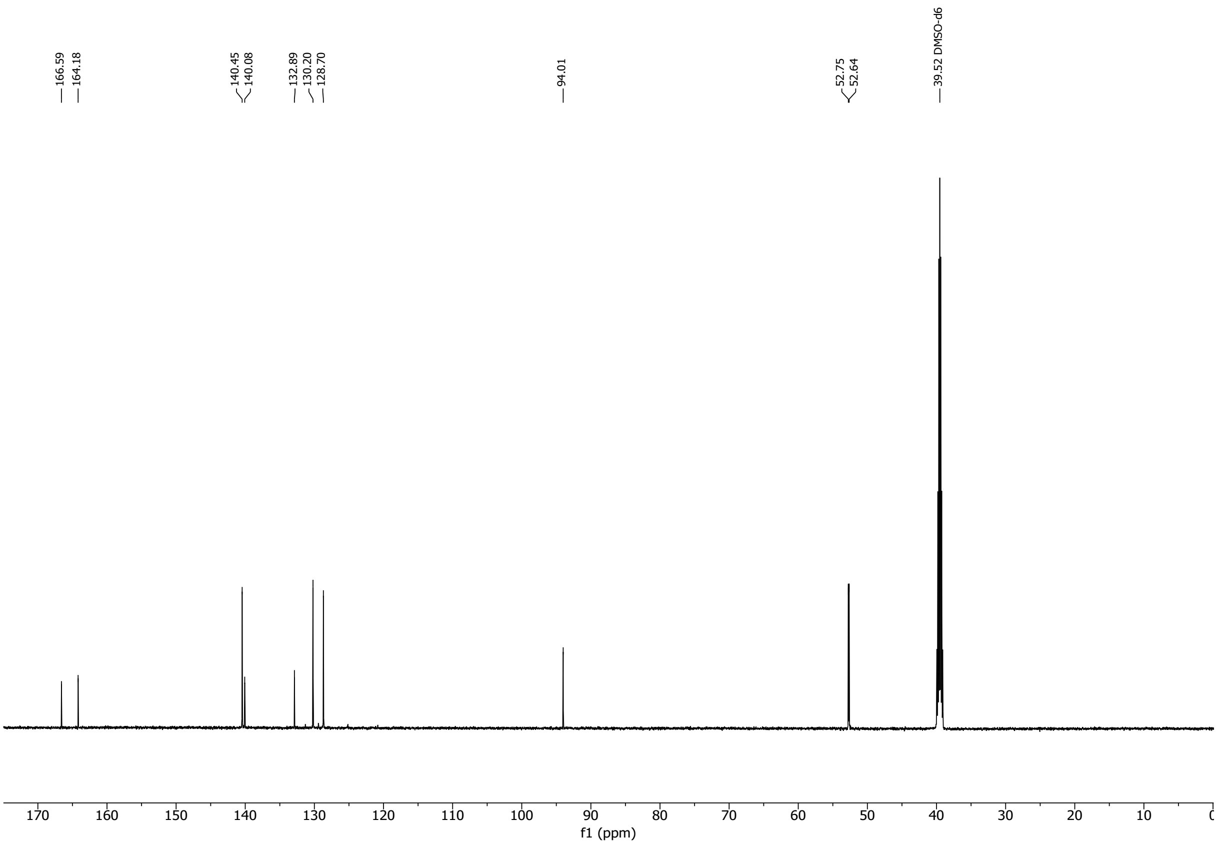

Figure S6. ^{13}C NMR spectrum of DMT-I (151 MHz, DMSO-d_6, 307 K).

2-Iodoterephthalamide (DMA-I)

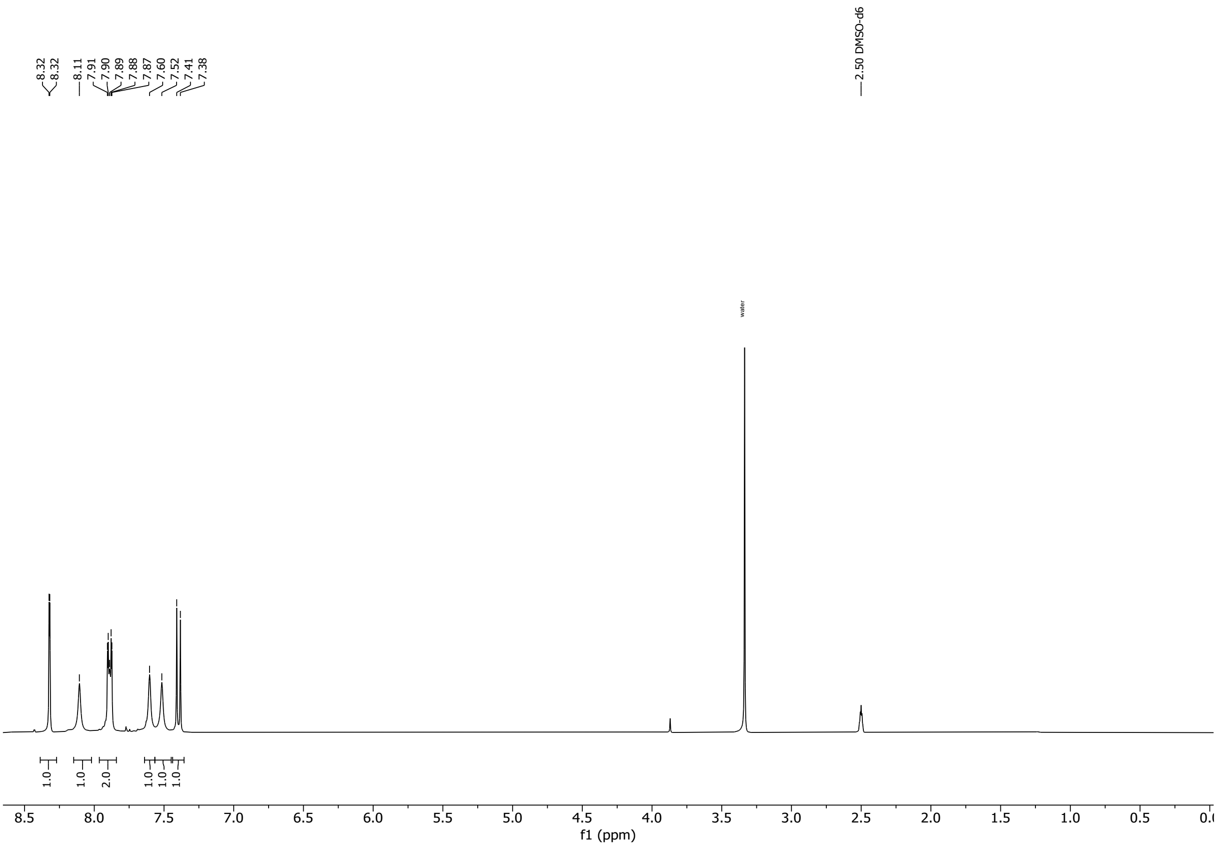

Figure S7. ^{1}H NMR spectrum of DMA-I (300 MHz, DMSO-d_6, 298 K).

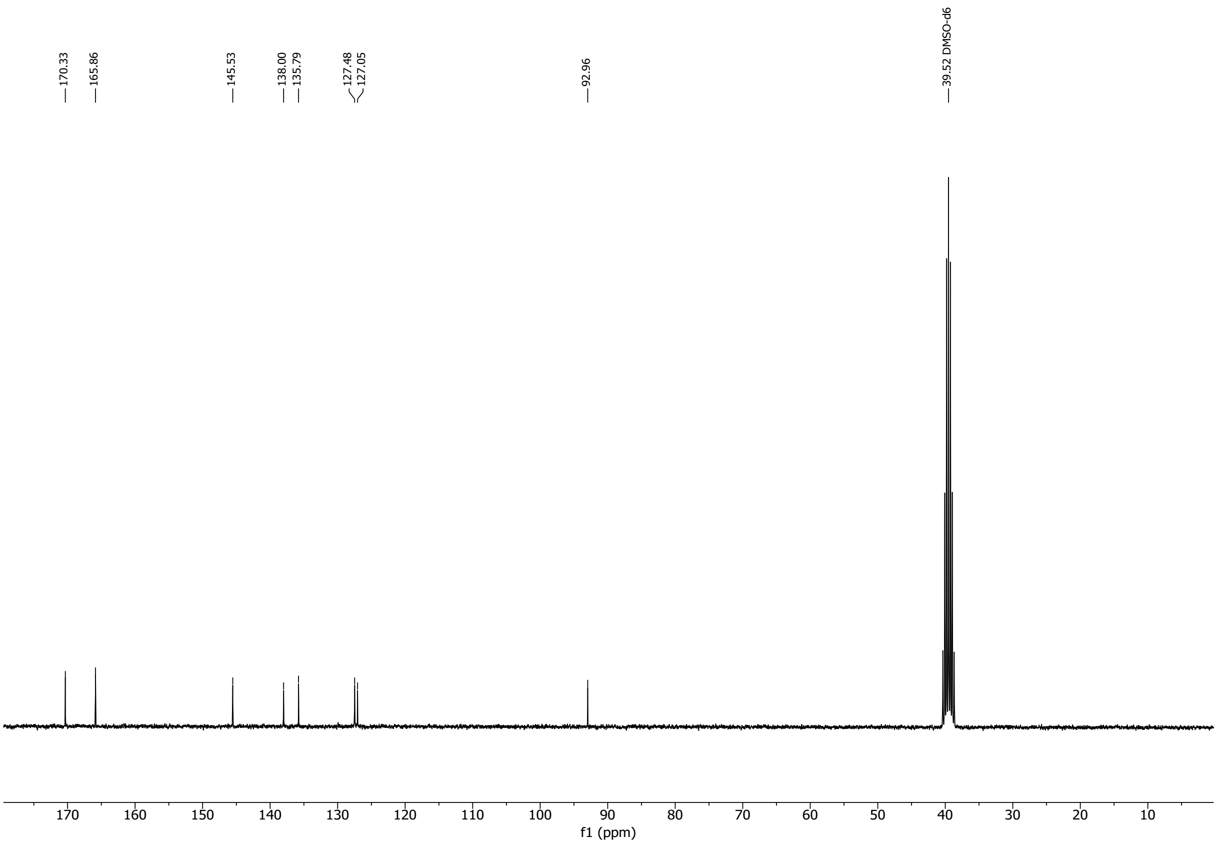

Figure S8. ^{13}C NMR spectrum of DMA-I (75 MHz, DMSO-d_6, 298 K).

2-Iodoterephthalnitrile (DCN-I)

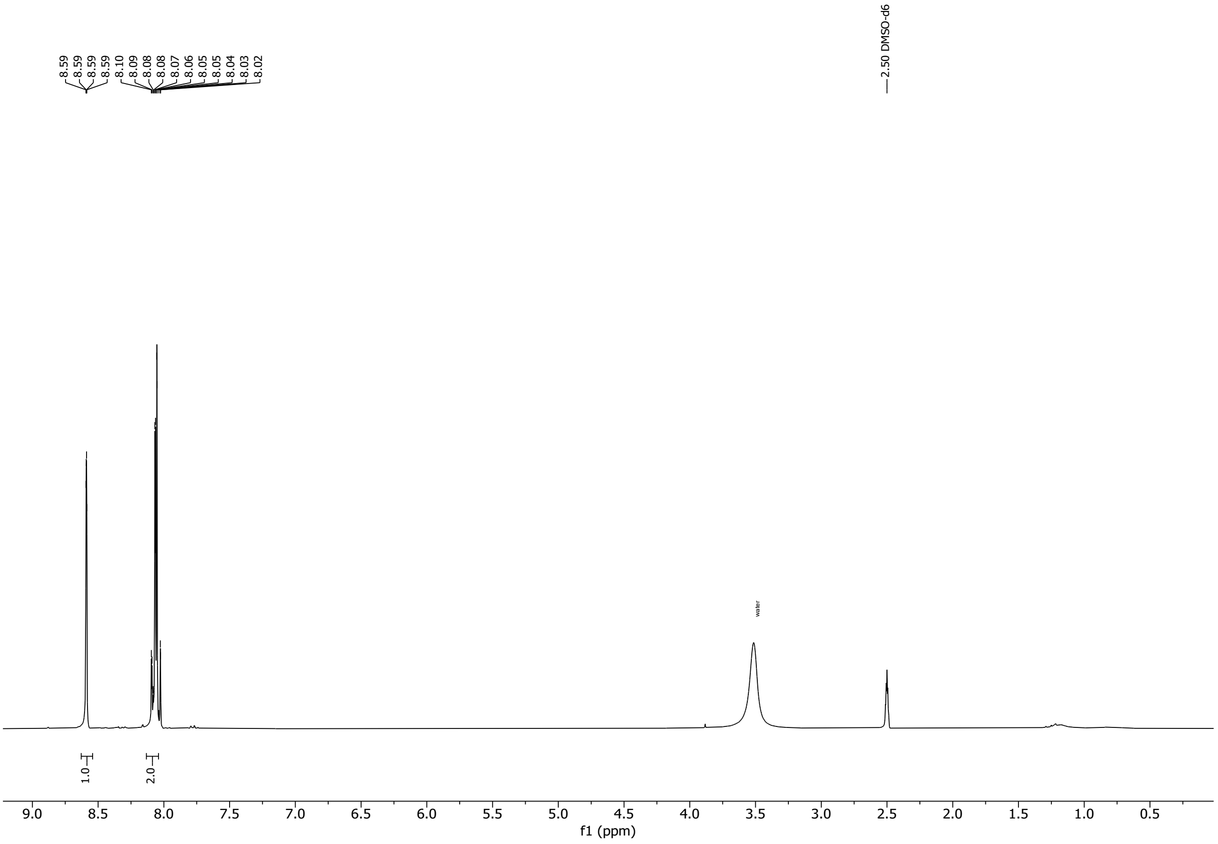

Figure S9. ^{1}H NMR spectrum of DCN-I (300 MHz, DMSO-d_6, 298 K).

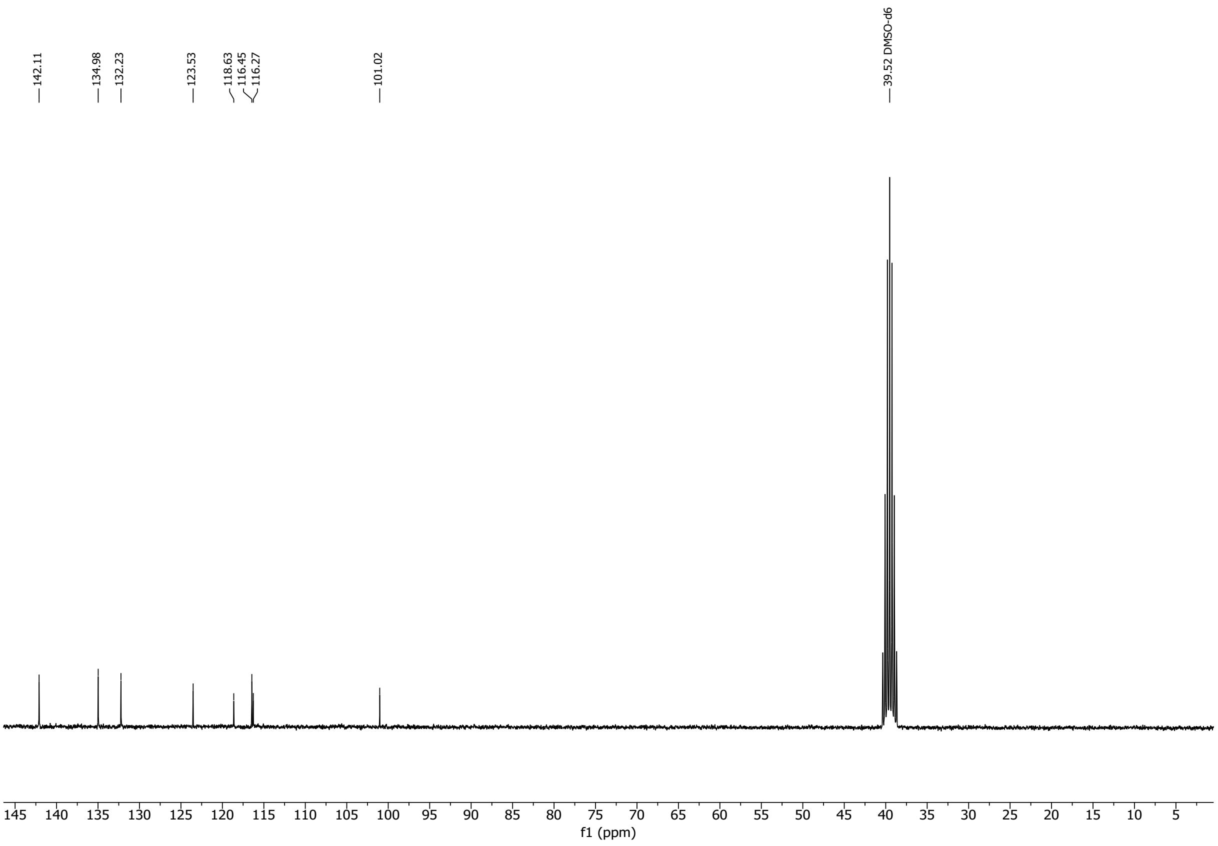

Figure S10. ^{13}C NMR spectrum of DCN-I (75 MHz, DMSO-d_6, 298 K).

4-Bromo-3-methyl-N,N-diphenylaniline (TAA-Br)

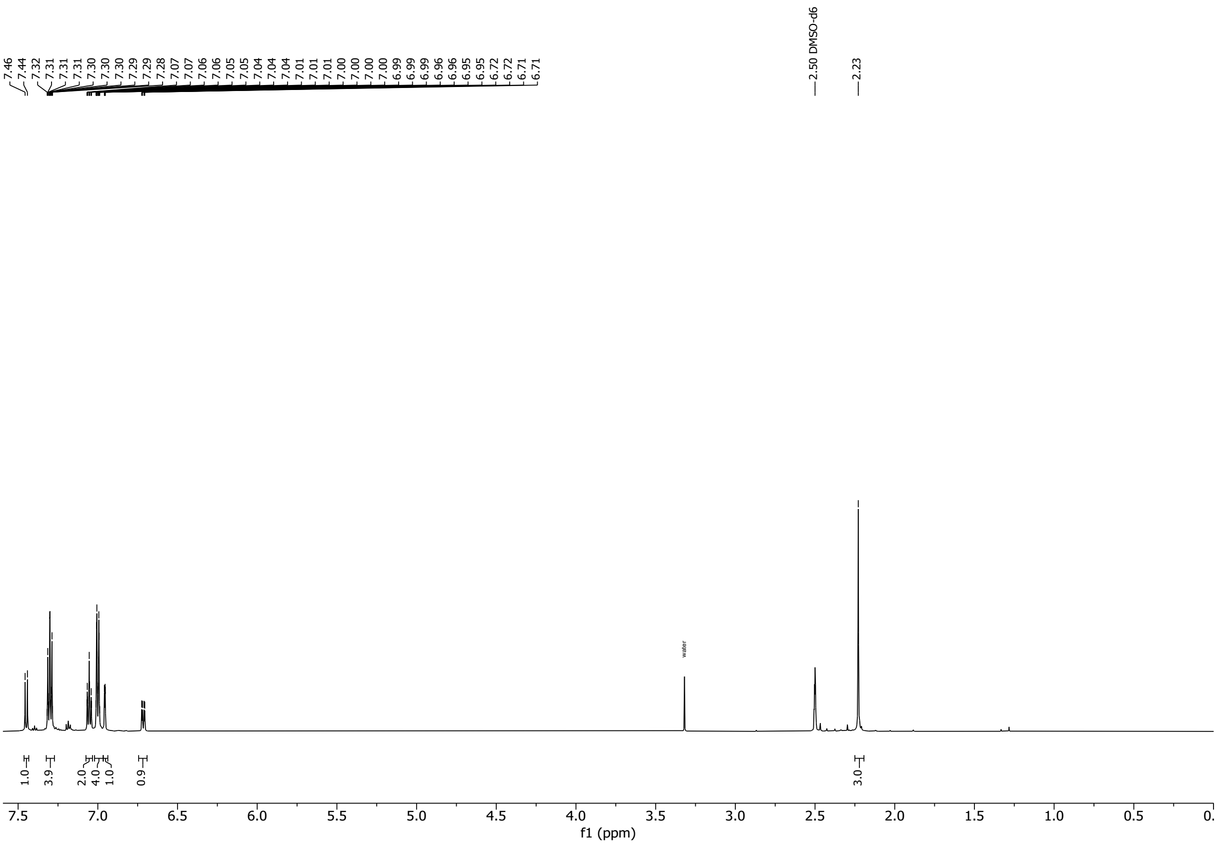

Figure S11. ^{1}H NMR spectrum of TAA-Br (600 MHz, DMSO-d_6, 298 K).

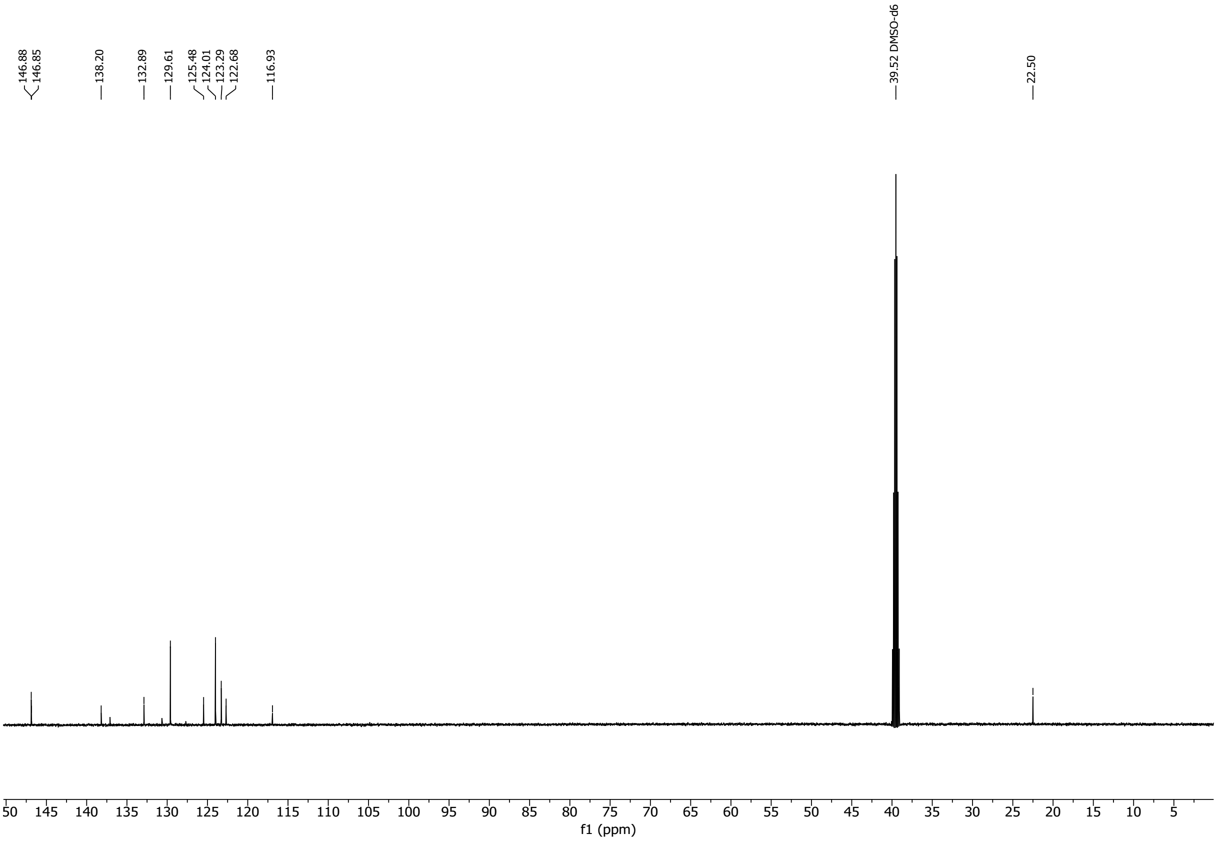

Figure S12. ^{13}C NMR spectrum of TAA-Br (151 MHz, DMSO-d_6, 298 K).

4'-(Diphenylamino)-2'-methyl-[1,1'-biphenyl]-2,5-dicarbonitrile (TAA-DCN)

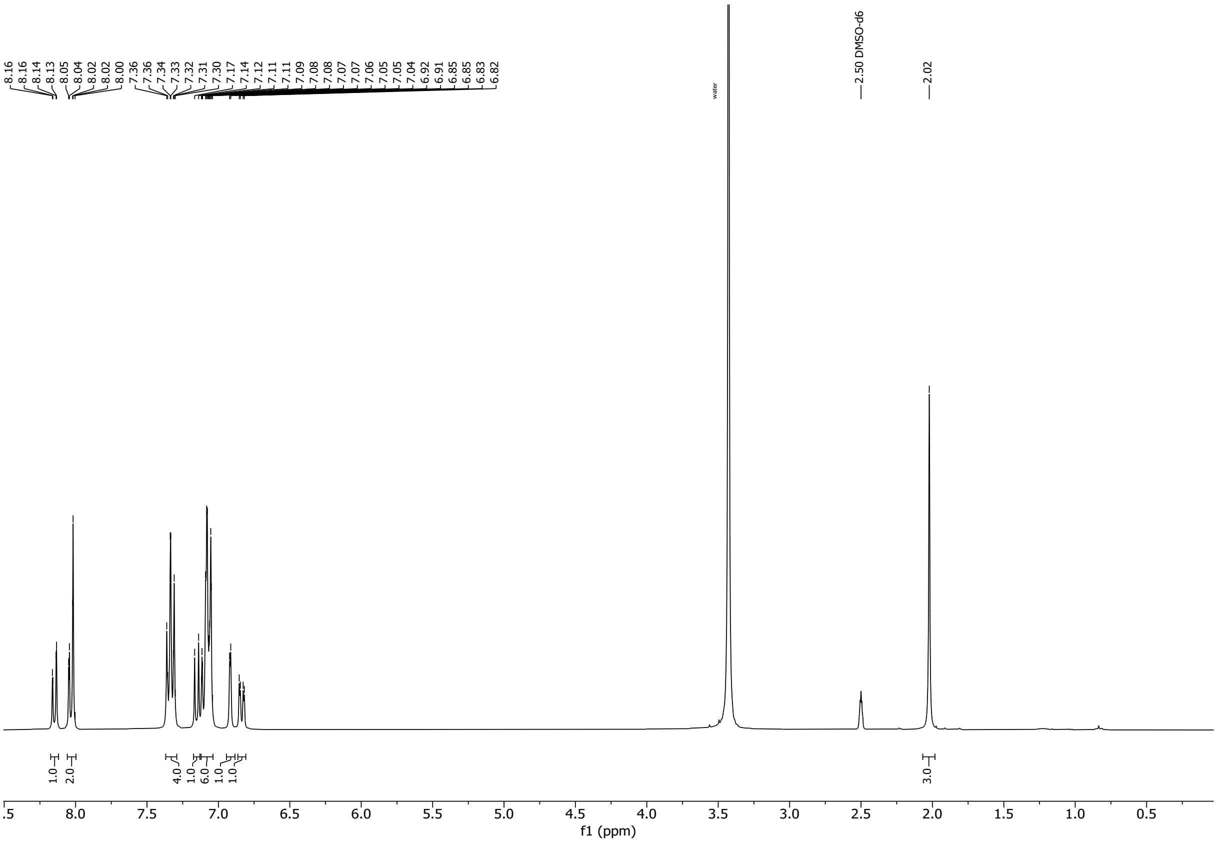

Figure S13. ^{1}H NMR spectrum of TAA-DCN (300 MHz, DMSO-d_6, 298 K).

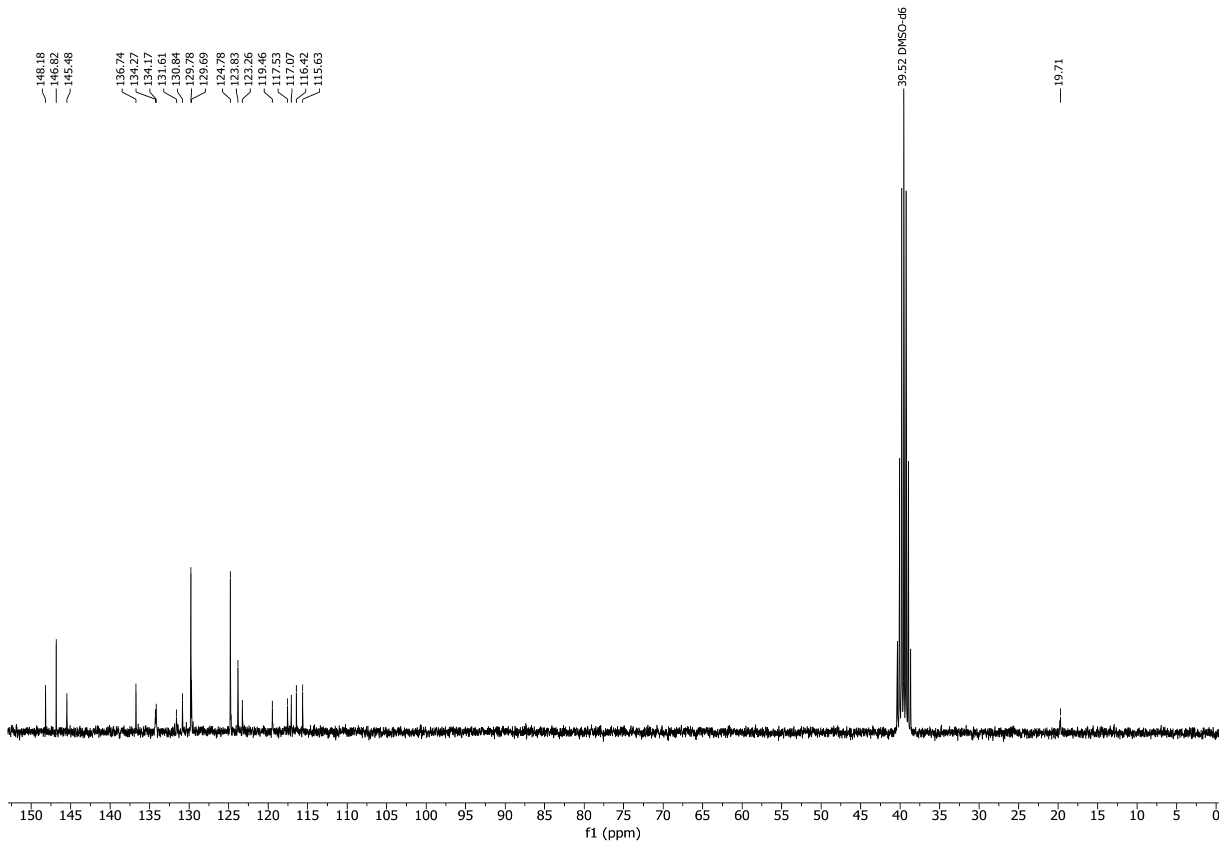

Figure S14. ^{13}C NMR spectrum of TAA-DCN (75 MHz, DMSO-d_6, 298 K).

S6. Characterization of the fsTA-NIR setup

Probe Spectrum

The fs-NIR probe light was obtained by supercontinuum generation with optimised conditions to achieve a broad spectral range in the NIR. Among the different conditions applied, the widest spectrum was achieved with an 8 mm thick sapphire crystal, and focusing with a 500 mm focal length. The fundamental light energy was finely tuned to enhance stability and prevent saturation in the detector (see dashed line in Figure S15 for saturation limit). The fundamental of the laser (centred at 800 nm) was filtered out by using a 2 mm thick long-pass filter RG850 (from SCHOTT), placed after the nonlinear crystal before the sample. Second order diffraction contributions were removed by another 1.1 mm thick long-pass filter, 20CGA-1000 (from Newport), placed inside the spectrograph. Typical continuum spectrum extended to ~1800 nm.

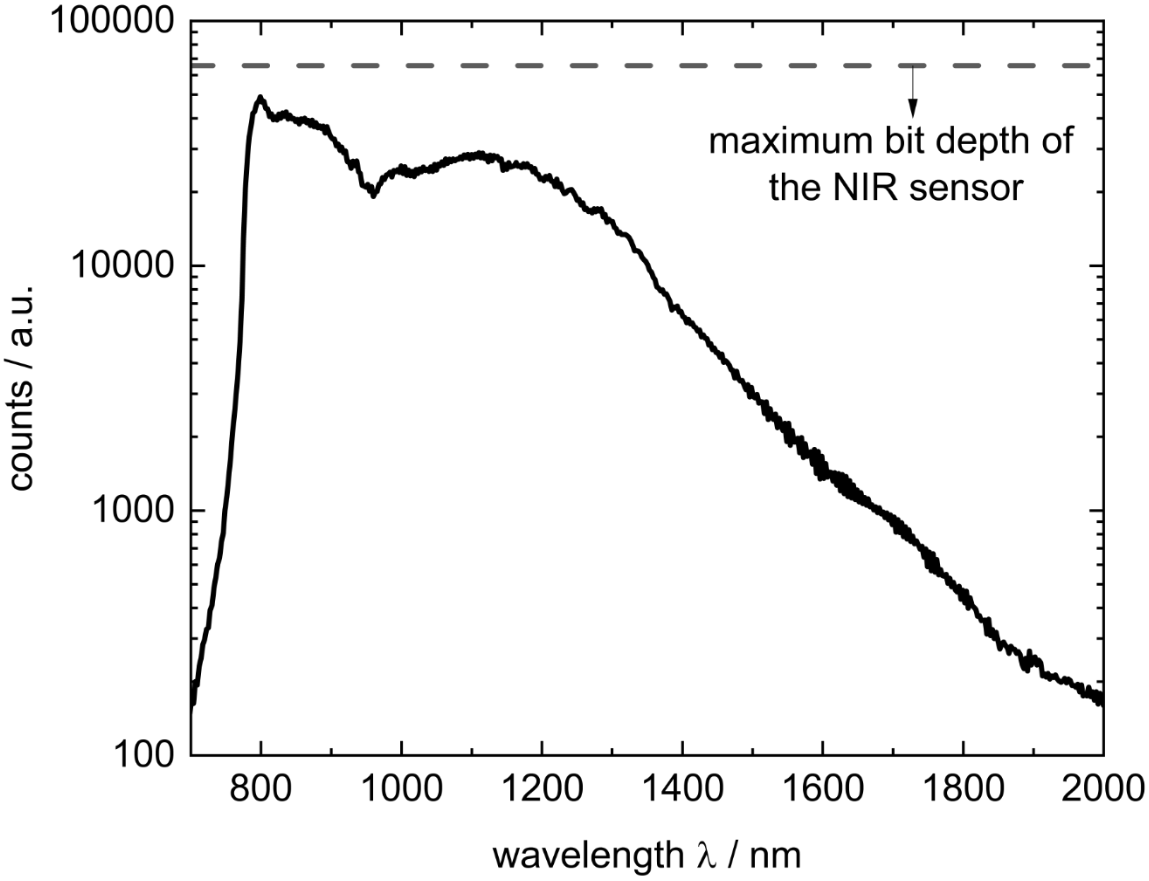

Figure S15. Typical supercontinuum spectrum generated using a sapphire crystal of 8 mm thickness and the fundamental (800 nm) of the Ti:Sa laser amplifier system. The spectrum is not corrected for the spectral sensitivity of the fsTA-NIR instrument. The “cut-off” at longer wavelengths is not due to the sensitivity of the detection system.

S7. Scaling procedure for the fsTA-NIR setup

The fsTA-NIR detector also has sensitivity in the visible spectral range. Thus, the transient absorption spectra recorded with the NIR setup could be overlapped spectrally with the ones recorded by the UV-Vis setup. For recording data with sensitivity in the visible range, fundamental light from the supercontinuum spectra was filtered out using a multiband filter (BG40, 1mm thick, from SCHOTT). To exemplify how the scaling procedure for the fsTA-NIR data was conducted, the comparison of both datasets for TAA-DCN solutions in cyclohexane is shown in Figure S16. The scaling factor was determined by the ratio (UV-Vis/NIR) of the maximum values of the transient absorption spectra at 100 ps. Here, a factor of 1.41 was obtained. This scaling procedure of the fsTA-NIR data accounts for any differences in excitation and probing conditions between both setups.

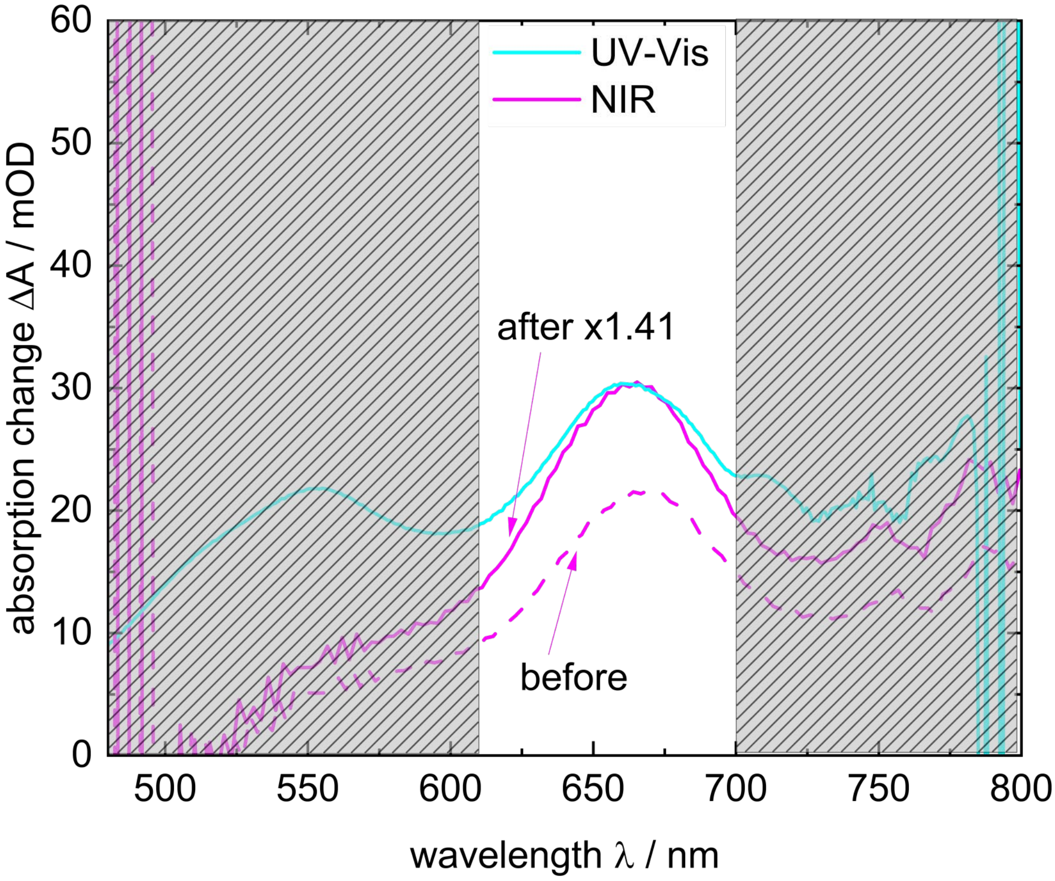

Figure S16. Comparison between the transient absorption spectrum obtained with the fsTA-NIR setup employing a multiband filter – BG40 – (pink lines) and the one obtained with the fsTA-UV-Vis (light blue line) setup for TAA-DCN dissolved in cyclohexane at 100 ps. The dashed pink line represents the transient spectrum before applying the scaling factor. The sample solutions were excited at 400 nm. The grey-dashed rectangles on the left (NIR) and right (UV-Vis) side represent the edges of the spectral ranges of the respective setups.

S8. Spectral and Temporal Features of the fsTA-NIR setup

The spectra and temporal capabilities of the implemented setup were tested by comparing measurements on β-carotene solutions in cyclohexane (c = 33 µM) with published ones[3-4]. According to the measurements reported in ref.[3], immediately after excitation at 480 nm, a strong absorption band at 990 nm (related to the $S_2 \rightarrow S_n$ transition) is observed. The system decays to the S_1 state within ≈ 0.19 ps, corresponding to the S_2 lifetime. A weak structured band around 1470 nm appears and decays with a time constant of 9.2 ps (related to the S_1 lifetime). Figure S17c) and d) show the fsTA-NIR measurements obtained with the new setup in comparison with a) and b) related to the reported values from literature. The spectral features measured in the new fsTA-NIR setup match the literature values. Also, the time dependence at 990 nm and 1470 nm matches the S_2 and S_1 lifetimes already reported with minor differences. To obtain the decay components of the time traces, the following trial functions (for 990 nm Equation (S1) and for 1470 nm Equation (S2)) were used:

$$S_{990\text{nm}}(t,\tau_{\text{cc}}) = IRF_{990}(\tau_{\text{cc}}) \otimes \left[A_1 e^{\left[-\frac{t}{\tau_1}\right]} + A_2 e^{\left[-\frac{t}{\tau_2}\right]} \right], \quad \textbf{(S1)}$$

$$S_{1470\text{nm}}(t,\tau_{\text{cc}}) = IRF_{1470}(\tau_{\text{cc}}) \otimes \left[A_3 e^{\left[-\frac{t}{\tau_3}\right]} \right], \quad \textbf{(S2)}$$

where $IRF(\tau_{cc}) \otimes$ stands for the convolution with the instrumental response function which was obtained by a Gaussian function with $\tau_{cc} = 0.2 - 0.3$ ps (FWHM, obtained for 990 nm and 1470 nm), and the fit yields time constants of τ_1 = 0.17 ps and τ_2 = 7.6 ps (for 990 nm) and τ_3 = 10.2 ps (for 1470 nm).

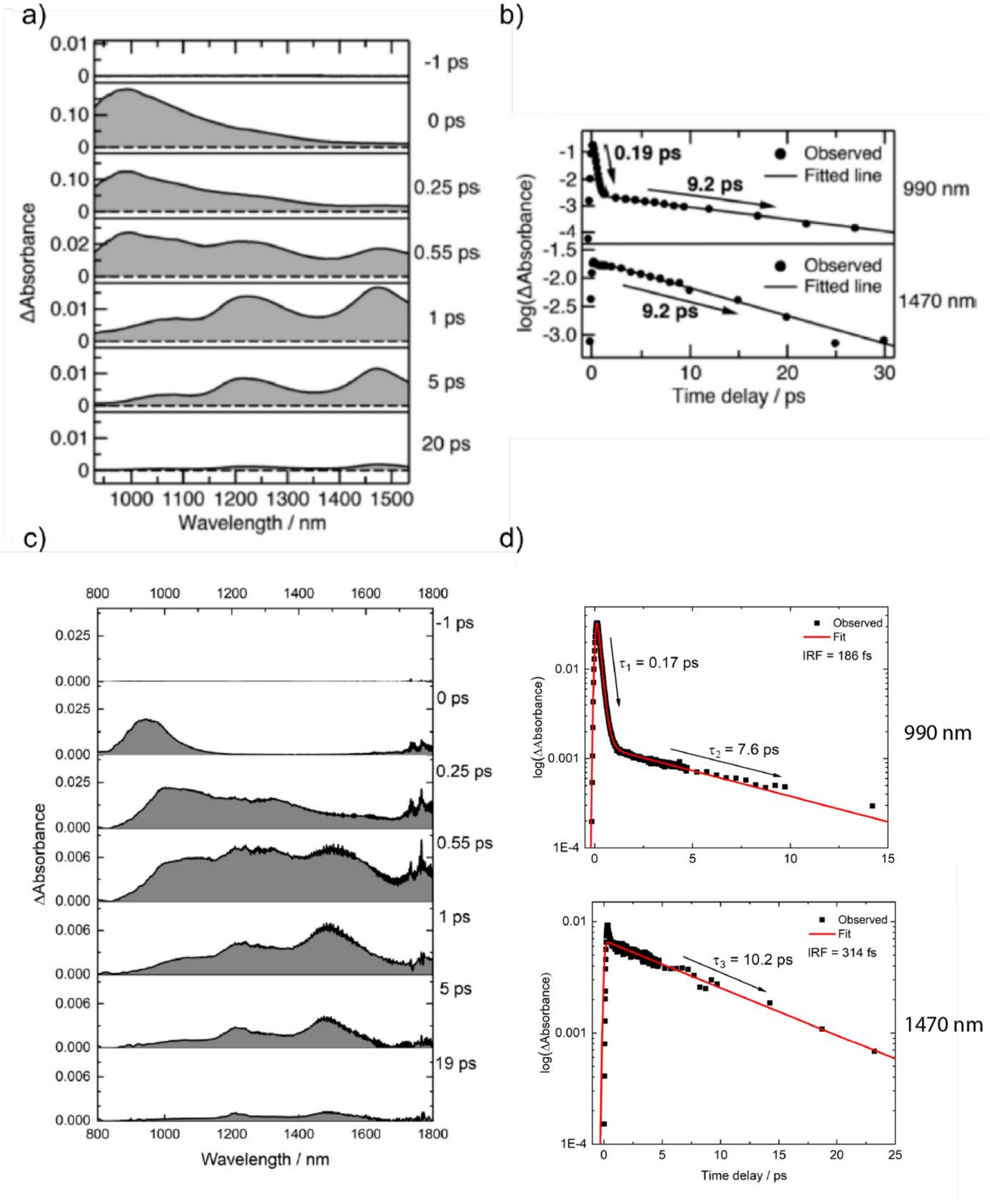

Figure S17. fsTA-NIR measurements on β-carotene in cyclohexane; transient absorption spectra shown in a), and time traces in b) due to excitation at 480 nm from literature ref.[3], and transient absorption spectra shown in c), and time traces in d) due to excitation at 400 nm performed in the new NIR setup. Figures a) and b) adapted with permission from ref.[3]. Copyright © 2014 American Chemical Society.

References

[1] W. Haselbach, J. M. Kaminski, L. N. Kloeters, T. J. Müller, O. Weingart, C. M. Marian, P. Gilch, B. E. Nogueira de Faria, *Chemistry–A European Journal* **2023**, *29*, e202202809.

[2] G. A. Sommer, L. N. Mataranga-Popa, R. Czerwieniec, T. Hofbeck, H. H. Homeier, T. J. J. Müller, H. Yersin, *The Journal of Physical Chemistry Letters* **2018**, *9*, 3692-3697.
[3] T. Takaya, K. Iwata, *J. Phys. Chem. A* **2014**, *118*, 4071-4078.
[4] T. Takaya, M. Anan, K. Iwata, *Physical Chemistry Chemical Physics* **2018**, *20*, 3320-3327.
